# Hypoxia induced DNMT3B and SHP2 signaling promoted HCC via suppressing P53 and MYH11 protein expression

**DOI:** 10.3389/fonc.2026.1794481

**Published:** 2026-06-15

**Authors:** Hongxun Ruan, Wei Huang, Yanle Fang, Ling Xie, Xiaoning Qin

**Affiliations:** The Second Hospital of Hebei Medical University, Shijiazhuang, China

**Keywords:** AKT, angiogenesis, CBFβ-MYH11, DNMT3B, hepatocellular carcinoma, HIF1α, hypoxia, SHP2

## Abstract

**Objective:**

This work aims to analyze the intricate process via which CBFβ-MYH11 facilitates the suppression of hepatocellular carcinoma in hypoxic environments.

**Methods:**

Three independent HCC cohorts including TCGA-LIHC, GSE14520 and ICGC LIRI-JP were enrolled. Gene correlation analysis, hypoxia-based expression comparison and prognostic stratification analysis were performed. A composite HIF1A/AKT/SHP2/DNMT3B/P53/MYH11 axis score was established to evaluate clinical prognostic value. Bioinformatics analysis was utilized to examine differentially expressed genes in hepatocellular carcinoma. In nude mouse models, tumor volume, tumor cross-sectional area, methylation levels, protein expression, vascular development, scratch assay, invasion assay, chromatin immunoprecipitation, and co-immunoprecipitation were quantitatively assessed. We used methods including HE staining, immunohistochemistry, phosphor-PCR, Western blot, angiogenesis tests, migration assays, and invasion assays.

**Results:**

Multi-cohort analysis revealed a stable negative correlation between DNMT3B and MYH11. High HIF1A expression was accompanied by DNMT3B upregulation and MYH11 silencing. Patients in the high axis score group exhibited markedly shorter overall survival, and the axis score served as an independent adverse prognostic risk factor. Bioinformatics analysis reveals MYH11-specific expression in hepatocellular carcinoma. There were clear differences between Huh-7-NC-CBFβ-MYH11 and Huh-7-OE-CBFβ-MYH11 when they were compared. Huh-7-OE-CBFβ-MYH11 showed a smaller tumor volume, less vascular density, fewer invasion cells, a shorter migration distance, and less vascular development. The expression of MMP2, VEGF, and HIF1α showed a decrease, while RUNX1 expression increased. The introduction of an AKT inhibitor in hypoxic conditions resulted in a more pronounced increase in P53 and CBFβ-MYH11 protein expression compared to the SHP2 inhibitor. AKT inhibitors eliminated differences in DNMT3B, HIF1α, and MMP2 expression between normoxia and hypoxia.

**Conclusion:**

In hypoxic conditions, CBFβ-MYH11 regulates the AKT/DNMT3B/SHP2 pathway to modulate variations in P53 expression, ultimately hindering the progression of hepatocellular carcinoma.

## 1 Introduction

Hepatocellular carcinoma (HCC) is the sixth most common cancer worldwide, and its morbidity and mortality rates are among the highest in the world. Although HCC can be treated with liver transplantation, interventional therapy, catheter arterial embolization, etc., the resulting tumor hypoxia leads to resistance to radiotherapy and chemotherapy and a poor prognosis. Therefore, understanding the underlying molecular mechanisms of HCC metastasis and finding new biomarkers and therapeutic drugs are of great significance for the prognosis and targeted treatment of HCC (1, 2). Common risk factors for HCC include viral infections such as hepatitis B/C virus infection, chronic liver disease such as fatty liver, alcohol abuse, and metabolic diseases including diabetes. Because its symptoms are so ambiguous, it’s typically hard to identify HCC. Early signs are mainly discomfort in the stomach, weight loss, and tiredness. Late signs might be jaundice, ascites, and fever (3). The goal of hepatocellular carcinoma screening and surveillance, which is commonly done using ultrasonography and alpha-fetoprotein (AFP), is to find hepatocellular carcinoma early on, which leads to greater cure rates and better results. Patients in the advanced stages of the disease cannot undergo major surgery. Patients with advanced hepatocellular carcinoma (HCC) have limited options in terms of availability and efficacy of treatment (4).

In this low-oxygen environment, the survivalist molecule AKT goes into protect mode, shielding cells and tissues from the dangers of oxygen deprivation. This activation of AKT is a survival mechanism by which cells can avoid dying from oxygen deprivation and to survive and grow (5). This pathway’s activation changes p-DNMT3B, which increases its effect and gradually changes the DNA methylation process, which in turn changes how cells work (6). However, the overexpression of p-DNMT3B is not entirely detrimental, as it possesses the pathogenic capacity to inhibit the tumor-suppressive function of P53, hence facilitating carcinogenesis and its subsequent progression (7). The molecular regulator SHP2 interacts with P53 in a complicated regulatory mechanism, affecting its activity (8). Moreover, SHP2 serves as a crucial regulator of AKT’s state, dictating its phosphorylation pattern and thus steering the course of the AKT signaling trajectory (9).

MYH11 (smooth muscle myosin heavy chain 11) is a crucial cytoskeleton-associated protein involved in cell migration, morphological maintenance and contraction regulation. Recent studies have verified that MYH11 is poorly expressed in hepatocellular carcinoma and acts as a tumor suppressor. Its downregulation facilitates the proliferation, invasion and angiogenesis of hepatoma cells, and is closely correlated with unfavorable clinical prognosis. Dysregulated MYH11 has also been reported to drive tumor progression in colorectal cancer, chondrosarcoma and other malignancies. Nevertheless, the expression regulation, epigenetic mechanism and downstream signaling pathways of MYH11 under hypoxic tumor microenvironment of liver cancer remain poorly understood.

CBFβ/MYH11 is a chromosomal abnormality present in acute myeloid leukemia resulting in a core binding factor ß subunit (CBFβ)/smooth muscle myosin heavy chain 11 (MYH11) fusion gene, which is caused by a pivotal inversion of chromosome 6, inv. (16) (p13.1q16) or t (16; 16) (p13.1; q16). Different breakpoints are observed at 16q22 of the CBFβ gene and 16p13.1 of the MYH11 gene. Thus, different CBFβ/MYH11 fusion genes were generated (10). It is commonly believed that CBFβ-SMMHC, a fusion protein encoded by CBFβ-MYH11, is a dominant negative repressor of RUNX1. CBFβ-SMMHC is thought to initiate leukemia by blocking normal haematopoietic differentiation through inhibition of RUNX1 function, and it can act as a transcriptional repressor by sequestering RUNX1 in the cytoplasm (11). The chimera CBFβ-MYH11, borne from a genetic mishap involving the amalgamation of the myosin-hardwiring MYH11 gene with the regulatory component CBFβ, emerges as a hallmark of chromosomal translocation, marking its presence with the signature t(16;16)(p13;q22). This recurrent translocation generates oncogenic implications, as evidenced by links to oncogenic processes in disparate cancers such as colorectal (12) and chondrosarcoma malignancies (13). CBFβ-MYH11, similar to the guardian P53, wields its power to alter the 121 dials of transcriptional control (14). However, how exactly CBFβ-MYH11 functions 122 in a hypoxic environment remains a mystery.

This article is a scientific trip for a close look at what CBFβ-MYH11 does in hepatocellular carcinoma in live things and test tubes. By doing thorough research, we want to build a strong theoretical base for the treatment plans that will fight this tough adversary of the liver.

## 2 Methods

### 2.1 Bioinformatics analysis

Firstly, STAR-counts data were obtained from TCGA-LIHC, normalized by log2(TPM + 1); GSE117361 (2 pairs of tumors/normal tissues) and GSE57957 (39 pairs of tumors/normal tissues) microarray data were downloaded synchronously, and the Robust Multi-array Average method was used to complete background correction, normalization and probe integration, and the Combat algorithm was applied to eliminate batch effects. Differential expression analysis was performed by limma package, and the screening criteria were |log2FoldChange|>1 and corrected p-value <0.05 (Benjamini-Hochberg method). The differential gene intersection of TCGA and GEO was imported into the ClusterProfiler package to carry out multilevel functional annotation: including the three dimensions of cellular components, molecular functions, and biological processes for gene ontology analysis, as well as KEGG pathway enrichment analysis (15, 16). Based on the TCGA-LIHC dataset, Spearman correlation analysis was used to reveal the covariation relationship between differential genes, and visualization and presentation of volcano plots and heat maps were achieved by ggplot2. Finally, the key gene expression patterns were verified in the GSE57957 dataset to ensure the reliability of the research results.

### 2.2 Cell culture and materials

Human normal hepatocyte cell line LO-2 cells and hepatocellular carcinoma Huh-7 cells and HepG2 cells were purchased from Wuhan Procell Biotechnology Co., Ltd. The human hepatocellular carcinoma cells Huh7 with good growth status were placed in a 10 cm diameter ordinary Petri dish, with complete medium added. They were cultured in an incubator at 37 °C and 5% CO2, and the medium was replaced every 24 hours. When the cells reached 80%-90% confluency, they were passaged. During passaging, the old medium in the dish was discarded, and the cells were washed 1–2 times with sterilized PBS buffer. After that, a solution of 0.25% EDTA trypsin was added to break down the cells. The cells were looked at with a light microscope. As soon as the cell form changed to a sphere and some of it fell off, the 0.25% EDTA trypsin was thrown away. Then, 4 mL of DMEM complete media was added, and the cells were gently blown off and mixed thoroughly. The cells were then split up in a 1:2 ratio.

We tossed away Huh7 cells that were in the logarithmic growth phase from the DMEM medium and washed them with sterilized PBS buffer one to two times. After that, a 0.25% EDTA trypsin digestion solution was added to break down the cells. The cells were looked at under a light microscope, and when they started to round out and come apart a little, the trypsin digestion was stopped by adding 2 mL of DMEM medium. The cells were gently blown down, transferred into a 15 mL centrifuge tube, and centrifuged at 1000 rpm for 5 minutes. The supernatant was discarded, and 3–4 mL of PBS was added for mixing to wash away the residual DMEM medium. The cells were centrifuged again at 1000 rpm for 5 minutes, and the supernatant was discarded. Several milliliters of prepared sphere-forming medium were added to the centrifuge tube. The cell concentration was counted using a hemocytometer, and 5.0×10^6 cells were added into a 10 cm diameter ultra-low attachment Petri dish. The cells were incubated under the conditions of 37 °C and 5% CO2. The size of the spheroids was observed daily, and serum-free sphere-forming medium was added each day. Over time, the cells gradually enriched into spheroids of different sizes, and when the morphology stabilized (approximately by the 4th day), the first generation of cells was passaged.

Cells in suspension culture were collected into a 15 mL centrifuge tube and centrifuged at 1000 rpm for 5 minutes. The old medium was discarded, and 1–2 mL of 0.25% EDTA trypsin was added. A pipette was used to repeatedly blow the cell spheroids until no visible spheroids remained (the suspension became clear). DMEM complete medium was added to terminate the trypsin digestion, and the cells were centrifuged at 1000 rpm for 5 minutes. The supernatant was discarded, and 3–4 mL of PBS was added for mixing to wash away the residual DMEM medium. The cells were centrifuged again at 1000 rpm for 5 minutes, and the supernatant was discarded. Several milliliters of sphere-forming medium were added, and the cells were transferred to a 10 cm diameter ultra-low attachment Petri dish for further cultivation. This was considered the second generation of Huh7 stem cell-like cells in suspension culture. The process was repeated every 4 days until the fourth generation of cells was obtained for experiments.

Low oxygen conditions were set at 37 °C in a 99% nitrogen 1% O_2_ environment for 3D culture. Akt inhibitor MK-2206 (5 nM), SHP2 inhibitor Batoprotafib (0.005 μM), P53 inhibitor Pifithrin-α (10 μM), and P53 activator Tenovin-1 (10 μM) were purchased from MCE.

### 2.3 Stable cell lines employing lentiviral transduction

All shRNA oligonucleotides were synthesized by Shanghai Sangyo and cloned into pLKO.1-TRC vector (Addgene #10878). The target sequences were as follows: sh-CBFβ-MYH11: 5’-GCA CCT GAA GTT CAT CAA GAA-3’ (targeting CBFβ exon5-MYH11 exon1 fusion region); sh-RUNX1: 5’-GCA AGA ACC AGC AGT ATG AAA-3’; sh-DNMT3B: 5’-AAC AAG ACT CGA AGA CGC A-3’; sh-NC: 5’-TTC TCC GAA CGT GTC ACG TAA-3’. In our state-of-the-art laboratory, we meticulously engineered and concentrated lentiviruses, namely LV-OE-NC, LV-OE-CBFβ-MYH11, LV-sh-NC, and LV-sh-CBFβ-MYH11, LV-sh-NC, LV-sh-RUNX1, DNMT3B-OE, and DNMT3B-KD. To prepare for lentiviral transduction, Huh-7 cells were plated at a density of 10^6 cells per well into 24-well plates approximately 18 hours in advance, ensuring optimal confluency of roughly 2×10^^5^ cells per well at the time of viral transduction. We meticulously established suitable virus concentration gradients, delicately added the virus to the cells in a 24-well plate, and incubated them at 37 °C within a serum-free environment to facilitate initial infection. 4 to 6 hours after infection, we replaced the medium with fresh medium containing serum to help the cells grow and heal as fast as possible. One day after virus exposure we initiated selection with puromycin. After tough 14 days selection process with puromycin, we succeeded to produce 4 stable cell lines which were still in their first 20 passages: Huh-7-OE-NC, Huh-7-OE-CBFβ-MYH11, Huh-7-sh-NC and Huh-7-sh-CBFβ-MYH11. All these lines were set up for further investigation and testing.

### 2.4 Tumorigenesis evaluation using nude mice models

We bought 24 male BALB/c-nu nude mice from Henan SKBS Biotechnology Co., Ltd. for a controlled biological investigation. They were all 6 weeks old and weighed between 18 and 20 grams. These animals were housed in a well controlled setting with stable temperatures of 25 ± 1 °C, a balanced humidity level of 50%-60%, and a structured 12-hour light/dark cycle. They also had free access to food and water. The Institutional Animal Ethics Committee fully approved the laboratory animal care and procedural methods used in this study, which closely followed the NIH criteria for humane animal research. We started making the tumor models by randomly dividing 24 BALB/c-nu nude mice into four groups, each with eight animals. The groups were named OE-NC, OE-CBFβ-MYH11, sh-NC, and sh-CBFβ-MYH11. Huh-7 cells from the right groups were harvested at logarithmic growth phase, normalized to the same concentration (1×10^7^ cells/ml) and gently suspended in 0.5 ml of matrix gel. After that, this cellular matrix was carefully injected into the right axillary region of each mouse’s subcutaneous tissue. The growth of the tumor was carefully watched, and calipers were used every day to obtain measurements of the tumor’s size as it changed over time. After a period of four weeks, when the tumor growth was expected to have reached a significant stage, the mice were carefully anesthetized using 3% isoflurane. The mice were killed with CO2, and the tumors were taken out surgically for more study. After 10 minutes of not breathing, nude mice were thought to have been successfully killed. And this study adhered to the Arrive standards. The Research Ethics Committee of the Second Hospital of Hebei Medical University gave this study the go light. After 28 days of injection, the tumors that were taken out were carefully photographed to record and compare how each treatment group in the research worked. The formula for tumor volume is V = 0.52 × a × b², where an is the longest diameter of the tumor and b is the diameter that is perpendicular to a. The 3Rs concept and the ARRIVE standards were followed for all procedures in this investigation.

### 2.5 HE staining

In the thorough procedure of histological inspection, our HE staining methodology started with the cautious cutting out of the tumor tissue. The samples started their preservation journey by going through a succession of ethanol solutions that slowly dehydrated them by taking water out of the tissues. After this, the frozen parts were swiftly but carefully fixed over a short gap of thirty seconds. They were then gently rinsed in water and placed into a warm hematoxylin solution (60 °C) for a full minute to receive the characteristic nuclear stain. After the bath, the pieces were rinsed again to remove excess hematoxylin. They were then dipped into a solution of 1% hydrochloric acid and ethanol for 3 sec and rinsed immediately in water. The pieces were put in a bluing solution for ten seconds, and then washed under running water for thirty seconds to improve the detail of the nuclear. The cells were stained with 0.5% eosin for 60 s and then rinsed immediately with distilled water. This is how the cytoplasm was detailed. Then the pieces were dipped in ethanol each time for 2 s starting with 80%, then 95% and finally 100%. That made cleaning easier. Finally the tissue slices were cleaned with xylene for 3 s. This was repeated two more times to ensure that the xylene had gone through all of the tissue. To complete the process, slides were assembled and fixed by a neutral adhesive to preserve the cellular features for further microscopic study. The size of the slices of tumor tissue was measured with the ImageJ program.

### 2.6 Immunohistochemistry

Paraffin sections were deparaffinized and rehydrated followed by antigen retrieval, blocking of endogenous peroxidase and incubation with primary and secondary antibodies. After DAB staining and hematoxylin counterstaining, the sections were dehydrated, cleared and mounted with neutral gum, and observed and photographed under the microscope.

### 2.7 Scratch assay

We utilized a 6-well plate to show how cells travel in a lab. Three horizontal reference lines were placed at the bottom of the plate to assist make scratches that are always the same. We plated logarithmic Huh-7 cells in the wells and allowed them to grow to 80% confluence. This resulted in a very dense cell environment. Using a 200 µl pipette tip, a vertical scratch was carefully made over the pre-marked lines to simulate a wound. The monolayer was then washed with PBS to remove any cells detached from the scratch area. Cells were treated and observed under an inverted microscope at the beginning (0h) and 48 hours after the scratch as intended for each experiment. “This inverted microscope made it easy to capture things and it also showed how cells migrated over time.

### 2.8 Invasion assay

To start we took BD Matrigel out of storage at -80 degrees and allowed it to thaw slowly at 4 degrees overnight. This was to see how invasive the cells are. We prepared a combination by combining 60 µl of Matrigel with 300 µl of serum-free medium. The mixture had to be kept at a cool temperature of 4 °C, ideally in an ice bath, to keep it from becoming uneven. In a careful manner, 100 µl of this gel matrix was spread evenly throughout three separate chambers in the upper chamber of the transwell system. After that, the setups were put in a 37 °C incubator for 4 to 5 hours, and the Matrigel layer was monitored every so often until a “white layer” appeared to show that it had solidified. After that, a serum-free medium wash was used to prepare the Matrigel, and then 100 µl of cell suspension was added. At the same time, the lower compartment was filled with 500 µl of medium that had 20% FBS added to it to function as a chemoattractant. The transwells were washed with PBS and then preserved with 5% glutaraldehyde at 4 °C for long-term storage after being in a 37 °C incubator for 20 to 24 hours. The invading cells could be seen after being stained with either crystal violet or Giemsa stain for ten minutes at room temperature. After staining, a cotton swab was used to carefully remove the cells from the top surface, and then a comprehensive microscopic investigation was done.

### 2.9 Angiogenesis assay

The method started with the simultaneous growth of Huh-7 stable cell lines and HUVEC for 48 h. The cell suspension of HUVECs was then evenly spread on the coated substrate of basic Matrigel in the culture tank. This made the cells spread evenly. The culture plate was then put in a controlled incubator to allow the cells to grow. The incubation period was 12 h at 37 o C and 5% CO 2. A microscope was then used to observe and record the formation of vascular systems, and photomicrography was used to capture the detailed micro-architecture. Use the Image J software to do the analytical step and look at the angiogenic patterns you saw in a quantitative way.

### 2.10 Methylation-specific PCR

Total DNA was extracted from whole blood using the DNA extraction kit (TIANGEN Biotechnology) and the concentration and purity of the extract were determined using a UV spectrophotometer. The extracted DNA (1.0 μg) was mixed with 7.0 μl of 3M NaOH in 100 μl of water (10ng/μl) and incubated at 50°C for 10 minutes. Subsequently, DNA modification reagent I was added and incubated at 50°C in the dark for 4 hours. The DNA was then modified with reagent III, followed by ethanol precipitation and purification steps. Methylation-specific PCR (MSP) was performed targeting the PNPLA3, FABP1 and MHY11 gene promoter regions as described, with PCR cycling conditions and primer sequences provided. The primer sequences were as follows: MYH11: forward methylation-specific primer: 5’-CGG GGA GTA GGA AGG TTA TTC-3’, reverse methylation-specific primer: 5’-ACA ACC CAA AAA AAA AAA CAA ACG-3’. Forward unmethylation-specific primer: 5’-GTT GGG GAG TAG GAA GGT TAT TT-3’, Reverse unmethylation-specific primer: 5’-AAC AAC CCA AAA AAA ACA CAC-3’. P53: Forward methylation specific primer: 5’-GTA GTT TGA ACG TTT TTA TTT TGG C-3’, Reverse methylation specific primer: 5’-CCT ACT ACG CCC TCT ACA AAC G-3’. Forward unmethylation specific primer: 5’-GTA GTT TGA ATG TTT TTA TTT TGG T-3’, Reverse unmethylation specific primer: 5’-GTA GTT TGA ATG TTT TTA TTT TGG T-3’.

### 2.11 ChIP q-PCR

Cells were cross-linked with 1% formaldehyde, and the chromatin was sheared into fragments of 200-1000bp. The chromatin fragments were immunoprecipitated with specific antibodies and the DNA-protein complexes were purified. After multiple washes, the DNA fragments were quantitatively analyzed by qPCR to determine the binding sites of the target protein in the genome.

### 2.12 RT-qPCR

Each group of cells was lysed with Trizol, total RNA was extracted, and the RNA was reversed to cDNA. The reaction system was configured according to the instructions, and 3 tubes were configured for each reverse transcription product, and 3 wells were made for each reverse transcription product to perform PCR amplification. Amplification conditions: pre-denaturation at 95 °C for 10 min; denaturation at 95 °C for 15 s, annealing at 60 °C for 30 s, extension at 72 °C for 30 s, 40 cycles. The Ct value of each reaction well was detected by PCR instrument, and the relative expression of the target gene was calculated according to the formula 2^-△△Ct^ with GAPDH as the internal reference. The sequences of primers were as follows: external primer for P53β: forward: 5’-GTC ACT GCC ATG GAG GAG CCG CA-3’, reverse: 5’-GAC GCA CAC CTA TTG CAA GCA AGG GTT C-3’; internal primer for P53β: forward: 5’-ATG GAG GAG CCG CAG TCA GAT-3’. reverse: 5’-TTT GAA AGC TGG TCT GGT CCT GA-3’; external primer of Δ133P53: forward: 5’-CTG AGG TGT AGA CGC CAA CTC TCT CTA G-3’, reverse: 5’-AGT CAG TCT GAG TCA GGC CCT TCT GTC-3’; internal primer of Δ133P53 internal primer: forward: 5’-GCT AGT GGG TTG CAG GAG GTG CTT ACA C-3’, reverse: 5’-CTC ACG CCC ACG GAT CTG A-3’; SHP2: forward: 5’-CTG GTG TGG AGG CAG AAA AC-3’, reverse: 5’-GGA CCA ACT CAG CCA AAG TG-3’; CBFβ-MYH11: forward: 5’-GGA TGG TAT GGG CTG TCT GG-3’, reverse: 5’-GAT GGG CCT TGC GTG ATA CT-3’; GAPDH: forward: 5’-GTC TCC TCT GAC TTC AAC AGC G-3’, reverse: 5’-ACC ACC ACC CTG TTG TTG GA-3’. ACC ACC CTG TTG CTG TAG CCA A-3’.

### 2.13 CO-IP

Total cell lysates were mixed with IgG (Abcam, ab172730) antibody and incubated overnight. Then 5 µL of agarose (A+G) was added and incubated for an additional 2 hours at room temperature. Composites were rinsed 3 times with PBS. The purified protein samples were analyzed by Western blot to detect specific protein interactions.

### 2.14 Western blot

Cultured cell specimens (5×10^6 cells) underwent dual cleanses utilizing PBS, were carefully detached from their cultivation substrate, and subsequently collected via a centrifugal procedure. We used 200 μl of RIPA buffer with 1 mM PMSF to stop proteolysis, and then we stored the collected cells at a cryogenic -80 °C. To separate the proteins from the cell lysates, they were mixed with 6*loading buffer and heated to 95 °C before being put into SDS-PAGE for electrophoretic separation. The amount of proteins needed varied from 80 to 120 μg depending on the experiment. After the proteins were separated, they were moved to a PVDF membrane and then interacted with primary antibodies at the best dilutions recommended by the manufacturer. The Western blotting regimen utilized a panel of discriminating antibodies, all sourced from Abtek Company: anti-MYH11 (CST, #79479, 1:1000), anti-CBFβ (CST, #62184, 1:1000), anti-p-AKT (Abcam, ab38449, 1:1000), anti-p-SHP2 (Abcam, ab62322, 1:1000), anti-p-DNMT3B (Abcam, ab321879, 1:1000), anti-P53 (Abcam, ab32049, 1:1000), anti-MMP2 (Abcam, ab92536, 1:1000), anti-VEGF (Abcam, ab32152, 1:1000), anti-HIF1α (Abcam, ab51608, 1:1000), and anti-GAPDH (Abcam, ab181602, 1:1000) as the housekeeping control. The ECL detection platform (Amersham Pharmacia Biotech) made it possible to see individual protein bands using chemiluminescence, while the Tanon EL5000 system kept track of the correct exposures.

### 2.15 MYH11 methylation assay

To investigate the impact of the hypoxic microenvironment on the epigenetic regulation of MYH11, transcriptome sequencing data (HTSeq-FPKM) of liver hepatocellular carcinoma (LIHC) were downloaded from The Cancer Genome Atlas (TCGA) database. A hypoxia score for each tumor sample was calculated using the average expression method based on the classical Buffa hypoxia gene signature (containing 51 genes). Based on the median hypoxia score, LIHC tumor samples were stratified into a Hypoxia group (High) and a Normoxia group (Low). Subsequently, DNA methylation beta values of CpG probes located in the MYH11 promoter region (including TSS1500, TSS200, 5’UTR, and 1st Exon) were extracted, and the average methylation level for each sample was computed. The Wilcoxon rank sum test was employed to compare the differences in MYH11 promoter methylation levels between the two groups, with statistical significance set at P<0.05.

### 2.16 Validation using public external databases

To validate the clinical relevance and cross-cohort robustness of the Hypoxia–AKT/DNMT3B/SHP2–P53/MYH11 signaling axis, three independent public datasets of hepatocellular carcinoma (HCC) were enrolled in this study for external validation: (1) TCGA-LIHC cohort (n=366), including RNA-seq expression profiles and complete clinical prognostic data, obtained via the TCGAbiolinks R package;(2) GSE14520 cohort (n=221), including Affymetrix HG-U133A microarray expression profiles and supplementary clinical data, downloaded from the GEO database;(3) ICGC LIRI-JP cohort (GSE144269, n=203), including voom-normalized gene expression data.

Correlation analysis: Expression levels of six key molecules (HIF1A, AKT1, PTPN11, DNMT3B, TP53, MYH11) were extracted from each cohort. Spearman’s rank correlation coefficient matrix was calculated, and heatmaps were generated to visualize the direction and strength of correlations between genes.

Hypoxia subgroup comparison: Samples in each cohort were divided into Hypoxia-High and Hypoxia-Low groups based on the median expression of HIF1A. Wilcoxon rank-sum test was used to compare the differential expression of DNMT3B and MYH11 between the two groups, and the results were presented as boxplots.

Axis Score construction and prognostic analysis: Based on the preliminary molecular mechanisms, a composite axis score was constructed as follows:Axis Score = Z(HIF1A) + Z(AKT1) + Z(PTPN11) + Z(DNMT3B) – Z(TP53) – Z(MYH11),where Z represents the Z-score of normalized gene expression (subtracting the mean and dividing by the standard deviation). Patients were classified into Axis-High and Axis-Low groups according to the median Axis Score. Kaplan-Meier method was applied to plot overall survival (OS) curves, and log-rank test was used to evaluate the intergroup differences. Furthermore, univariate Cox proportional hazards model was performed to assess the association between Axis Score and OS, with hazard ratio (HR) and 95% confidence interval calculated.

All statistical analyses were performed using R software (version 4.x). P < 0.05 was considered statistically significant.

### 2.17 Statistical analysis

All experiments were performed with at least three independent biological replicates (*in vitro* experiments: n = 3 per group; *in vivo* mouse experiments: n = 8 mice per group). Experimental data are presented as the mean ± standard deviation (SD). The Shapiro–Wilk test was used to assess the normality of data distribution, and Levene’s test was applied to verify the homogeneity of variance. For comparisons between two groups, the unpaired two-tailed Student’s t-test was used to determine statistical significance if the data were normally distributed and variance was homogeneous; otherwise, the Mann–Whitney U test was employed.For comparisons among multiple groups, one-way ANOVA was performed first. If variance was homogeneous, Tukey’s *post hoc* test was used for pairwise comparisons; if variance was heterogeneous, Welch’s ANOVA combined with the Games-Howell *post hoc* test was applied.For experiments involving two independent variables, two-way ANOVA was conducted, followed by Šídák’s *post hoc* test for multiple comparisons.For all analyses involving multiple comparisons, P-values were adjusted using the Benjamini–Hochberg false discovery rate (FDR). A P value < 0.05 was considered statistically significant. All statistical analyses were performed using GraphPad Prism 9.0 software. Statistical significance was denoted as follows: *P < 0.05, **P < 0.01, and ns (not significant) for P ≥ 0.05.

## 3 Results

### 3.1 Bioinformatics analysis results

**Figure 1:**
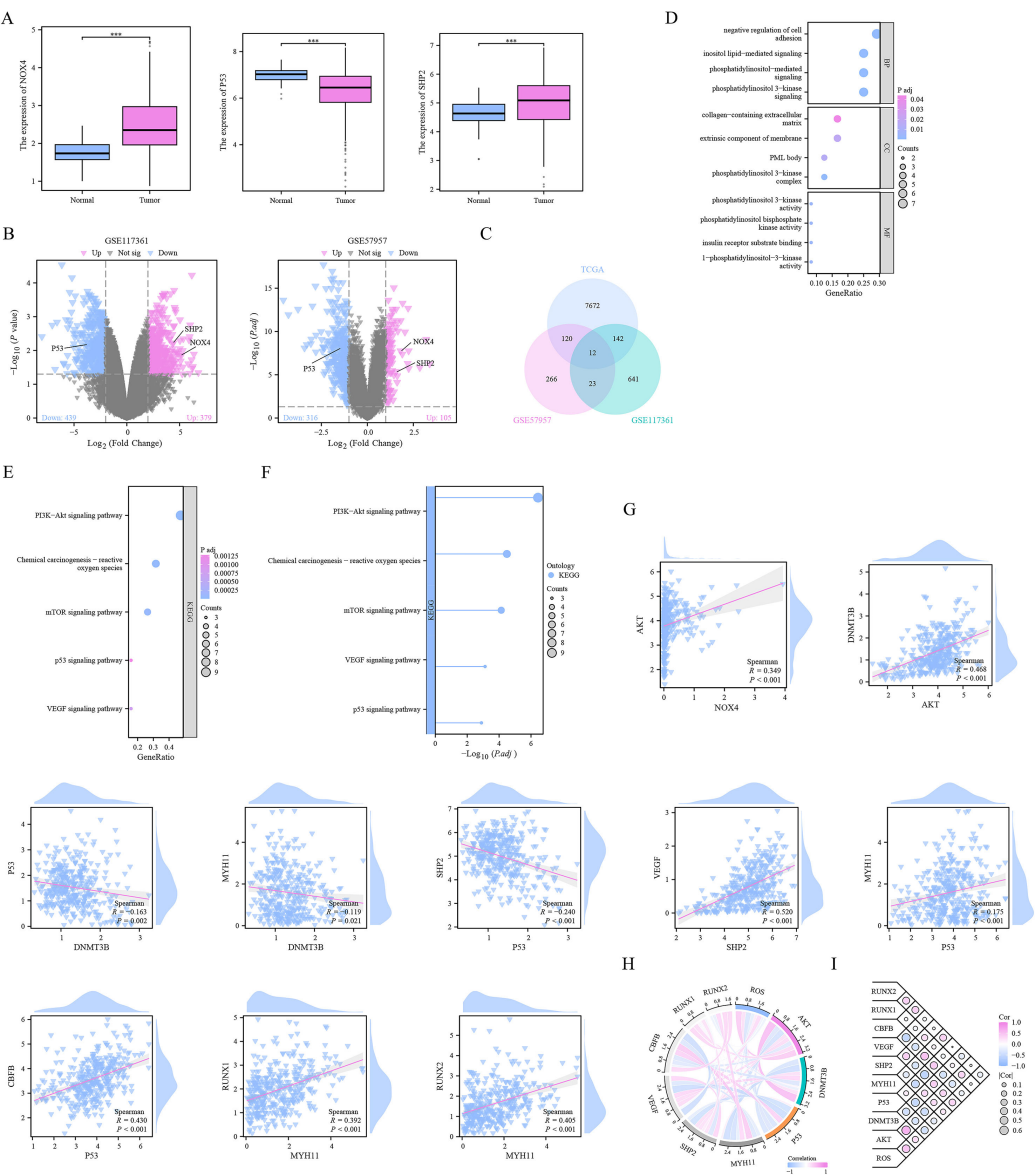
Bioinformatics analysis. **(A)** Distribution of ROS marker protein NOX4, TP53 and SHP2 expression in hepatocellular carcinoma tissues versus normal tissues in the TCGA-LIHC cohort; **(B)** Volcano plot of differentially expressed genes in GSE117361 and GSE57957; **(C)** Wayne diagram of GSE117361, GSE57957 and TCGA-LIHC differential gene intersection; **(D)** Core differential gene GO function enrichment bubble plot; **(E)** KEGG pathway enrichment bubble plot; **(F)** KEGG pathway enrichment lollipop plot; **(G)** Correlation scatter plot in TCGA-LIHC; **(H)** GEO dataset gene interaction network chord plot; **(I)** Heatmap of gene co-expression in GEO dataset.

Wilcoxon rank sum test based on the TCGA-LIHC cohort showed that ROS marker protein NOX4 and SHP2 were significantly highly expressed in hepatocellular carcinoma tissues, whereas P53 showed a significantly low expression profile (**Figure 1A**). Validation of the GSE117361 (818 differential genes: 379 up/439 down) versus GSE57957 (421 differential genes: 105 up/316 down) datasets revealed that ROS marker protein NOX4 and SHP2 were consistently highly expressed in tumor tissues, while P53 was systematically under-expressed (**Figure 1B**). Wayne plot analysis revealed a total of 12 core differential genes across the three datasets (**Figure 1C**), which were analyzed by ClusterProfiler Multiomics Enrichment Analysis GO analysis revealed that DEGs were expressed in cellular components such as phosphatidylinositol 3-kinase complex, PML body and extrinsic component of the membrane and other cellular components; DEGs were significantly enriched in 1-phosphatidylinositol-3-kinase activity, Phosphatidylinositol bisphosphate kinase activity and Phosphatidylinositol 3-kinase activity were significantly enriched in molecular functions; DEGs were significantly enriched in phosphatidylinositol 3-kinase signaling, phosphatidylinositol-mediated signaling and inositol lipid-mediated signaling (**Figure 1D**); KEGG pathway analysis revealed that DEGs were significantly enriched in PI3K-Akt signaling pathway, chemical carcinogenesis - reactive oxygen chemicals, mTOR signaling pathway, P53 signaling pathway and VEGF signaling pathway (**Figures 1E, F**); Spearman’s analysis revealed a strong positive correlation between ROS marker protein NOX4 and AKT in TCGA, suggesting that ROS may drive hepatocellular carcinoma progression by activating the AKT signaling axis; the positive correlation between AKT-DNMT3B positive correlation and DNMT3B-P53 negative correlation together constitute an epigenetic regulatory network; the multiple interactions of P53 with SHP2, MYH11 and CBFβ reveal its function as a core regulatory node; and the positive correlation of MYH11-RUNX1/2 and SHP2-VEGF suggests the synergistic activation of cell differentiation and angiogenic pathways (17, 18) (**Figure 1G**). Validated by correlation chordal plots and heatmap visualization, the GEO dataset showed a high degree of consistency with the TCGA analysis results, confirming the biological robustness of this molecular network (**Figures 1H, I**).

### 3.2 Elucidating the role of CBFβ-MYH11 in attenuating tumorigenicity of hepatocellular carcinoma cells

**Figure 2:**
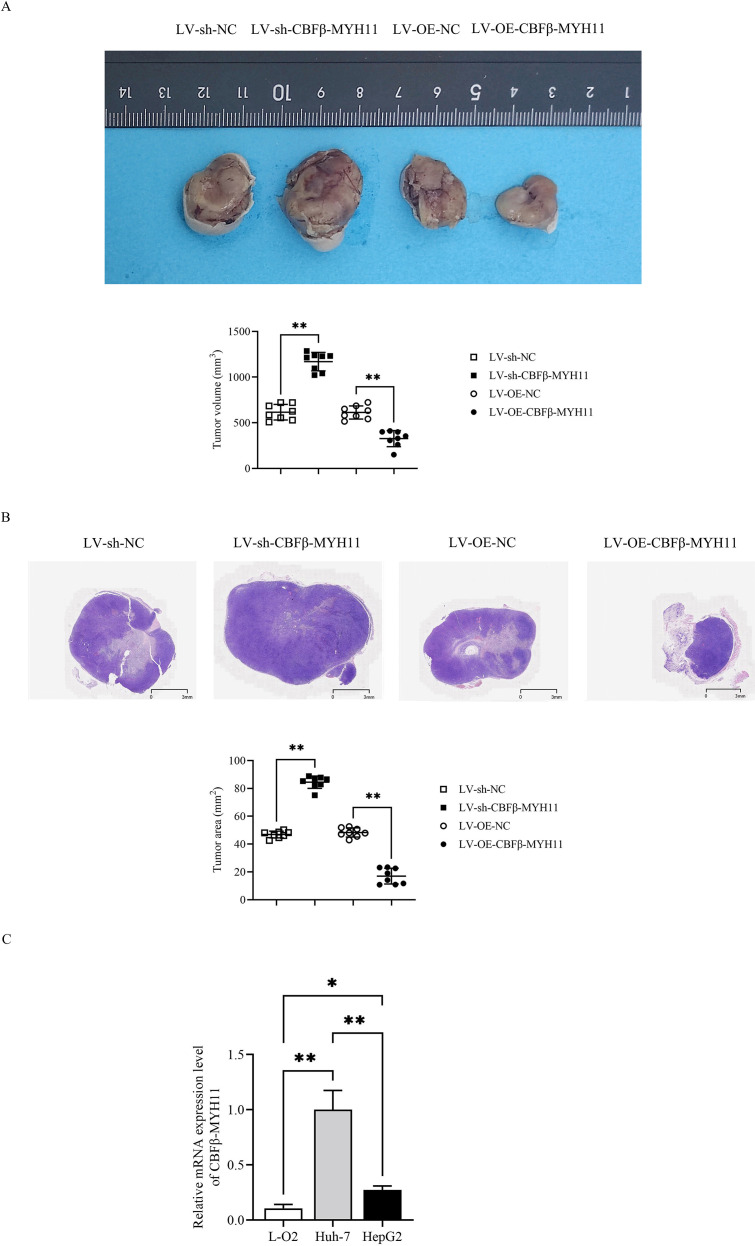
Suppression of tumor formation ability of hepatocellular carcinoma under CBFβ-MYH11 inhibition. **(A)** Graphs of the results of subcutaneous transplantation tumor experiments in nude mice in the LV-sh-NC group, LV-sh-CBFβ-MYH11 group, LV-OE-NC group, and LV-OE-CBFβ-MYH11 group, as well as the statistical graphs of tumor volumes; **(B)** Graphs of the results of HE staining experiments in the LV-sh-NC group, the LV-sh-CBFβ-MYH11 group, the LV-OE-NC group and the LV-OE-CBFβ-MYH11 group, as well as the statistical graphs of the area of tumor sections; **(C)** Relative mRNA expression levels of the CBFβ-MYH11 fusion gene in LO-2, Huh-7, and HepG2 cells were detected by reverse transcription quantitative polymerase chain reaction (RT-qPCR) assays. Data were expressed as mean ± standard deviation. N = 8; **P<0.01.

In an exhaustive investigation designed to elucidate the influence of CBFβ-MYH11 on the tumorigenic propensities of Huh-7 hepatocellular carcinoma cells, an experimental model was established in which Huh-7 cells, variably transfected with constructs for knockdown (LV-sh-CBFβ-MYH11) and overexpression (LV-OE-CBFβ-MYH11) of the CBFβ-MYH11 gene, as well as their corresponding negative controls (LV-sh-NC, LV-OE-NC), were xenografted subcutaneously into immunodeficient nude mice. Upon reaching a marked time point of 30 days post-inoculation, the mice were relieved of their tumor burdens which were subsequently documented through photography (**Figure 2A**).

HE staining results showed that tumor volume was significantly reduced in the cohort of mice treated with overexpressed constructs. Compared with the LV-OE-NC group, the tumor area of the LV-OE-CBFβ-MYH11 group was statistically significantly reduced. The tumor area of mice with weakened expression of CBFβ-MYH11 by LV-sh-CBFβ-MYH11 intervention was significantly larger than that of the negative control group (**Figure 2B**). Furthermore, to clarify whether the CBFβ-MYH11 fusion gene is endogenously present in hepatocellular carcinoma (HCC), we performed reverse transcription quantitative polymerase chain reaction (RT-qPCR) assays. The results demonstrated that the relative mRNA expression level of CBFβ-MYH11 was significantly higher in Huh-7 and HepG2 cells than in LO-2 cells. Additionally, the relative mRNA expression level of CBFβ-MYH11 in Huh-7 cells was markedly higher than that in HepG2 cells (**Figure 2C**).

The above results indicated that CBFβ-MYH11 attenuated the tumorigenicity of hepatocellular carcinoma cells.

### 3.3 Mechanistic analysis of CBFβ-MYH11 inhibition in hepatocellular carcinoma

A comprehensive exploration was undertaken to delineate the mechanisms by which CBFβ-MYH11 influences hepatocellular carcinoma growth, with a particular focus on the critical roles of hypoxia and angiogenesis within tumor progression. Proteins were meticulously extracted from tumor tissues harvested from stable Huh-7 cell lines and subjected to Western Blot analysis to unravel the molecular underpinnings.

**Figure 3:**
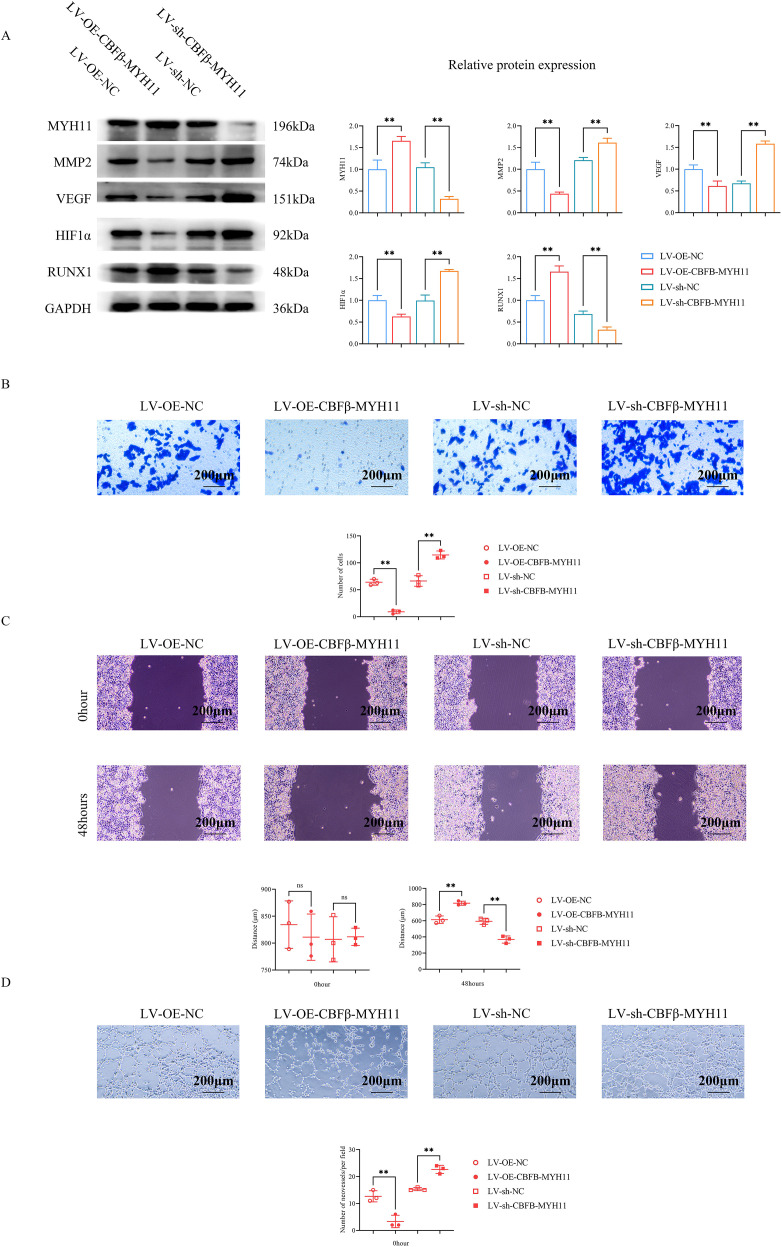
Inhibition of migration, invasion, and angiogenesis ability of hepatocellular carcinoma by CBFβ-MYH11 through suppression of MMP2, VEGF, and HIF1α. **(A)** Western blot experiments were performed to detect protein banding plots of MYH11, MMP2, VEGF, HIF1α, and RUNX1 as well as statistical plots of relative protein expression in the LV-sh-NC group, LV-sh-CBFβ-MYH11 group, LV-OE-NC group, and LV-OE-CBFβ-MYH11 group. GAPDH as control protein; **(B)** Graphs of experimental results of Transwell assay to detect the cell migration ability of LV-sh-NC group, LV-sh-CBFβ-MYH11 group, LV-OE-NC group and LV-OE-CBFβ-MYH11 group as well as statistical graphs of the number of migrated cells; **(C)** Plots of experimental results of cell scratch assay to detect cell migration ability of LV-sh-NC group, LV-sh-CBFβ-MYH11 group, LV-OE-NC group and LV-OE-CBFβ-MYH11 group as well as statistical plots of cell spacing; **(D)** Graphs of the experimental results of Angiogenesis experiment to detect the angiogenic ability of LV-sh-NC group, LV-sh-CBFβ-MYH11 group, LV-OE-NC group and LV-OE-CBFβ-MYH11 group, as well as statistical graphs of the number of blood vessels. Data were expressed as mean ± standard deviation. N = 3; **P<0.01; ^ns^P>0.05: There was no significant difference.

The experimental revelations from Western Blots indicated that relative to the LV-OE-NC group, enhanced expression of MYH11 and RUNX1 were observed in the LV-OE-CBFβ-MYH11 group, with concurrent attenuation in the expression of key players in angiogenesis, namely, MMP2, VEGF, and HIF1α. In stark contrast, LV-sh-CBFβ-MYH11 demonstrated a significant reduction in MYH11 and RUNX1 protein levels, with a concomitant surge in MMP2, VEGF, and HIF1α expression when juxtaposed with the LV-sh-NC group (**Figure 3A**).

Building on the *in vivo* findings, several *in vitro* functional assays were regulated to corroborate the impact of MYH11 on cellular behaviors pertinent to malignancy. Transwell migration assays revealed a marked decrement in the traversal of Huh-7 cells through the matrix in the LV-OE-CBFβ-MYH11 group as compared to the control, while an increased migration phenomenon was pronounced in LV-sh-CBFβ-MYH11 (**Figures 3B, C**). Furthermore, the wound healing assays demonstrated a significant persistence of scratch width at 48 hours post-induction in the LV-OE-CBFβ-MYH11 group, whereas an accelerated closure was evident in cells with reduced MYH11 expression (**Figures 3B, C**).

Angiogenesis assays lent credence to this hypothesis, where substantial reductions in neovascularization were observed in the LV-OE-CBFβ-MYH11 group compared to the control. Conversely, an upregulation in angiogenesis was detected in the LV-sh-CBFβ-MYH11 iterations (**Figure 3D**).

Synthesizing these results, it was posited with confidence that CBFβ-MYH11 exerts a repressive impact on the migration, invasion, and angiogenesis capabilities of hepatocellular carcinoma, primarily by the downregulation of pivotal angiogenic mediators: MMP2, VEGF, and HIF1α.

### 3.4 AKT and SHP2 under hypoxic conditions collectively influence the methylation changes of P53 and CBFβ-MYH11 mediated by DNMT3B

**Figure 4:**
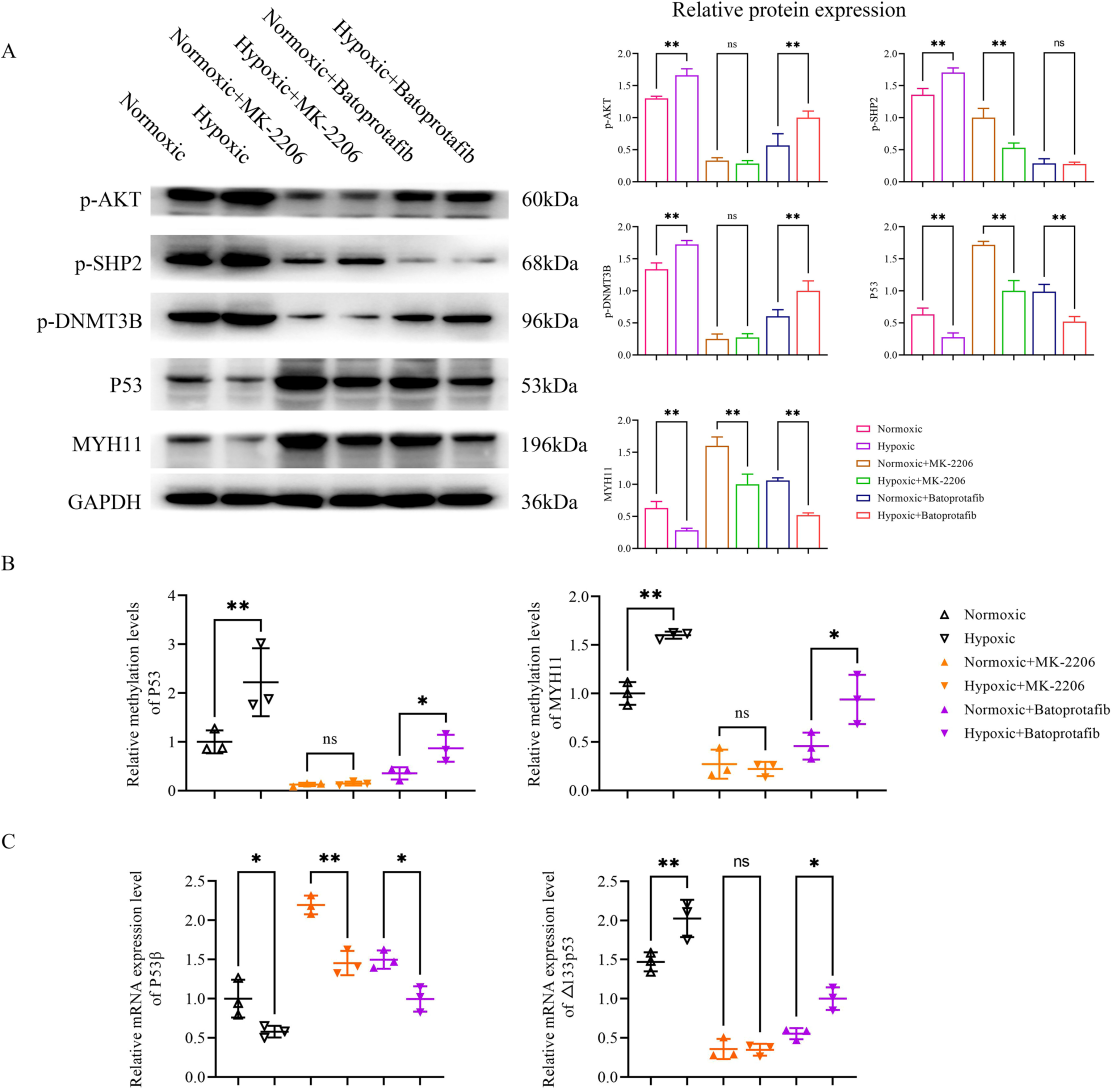
Regulation of hepatocellular carcinoma progression by CBFβ-MYH11 under hypoxic conditions through AKT and SHP2 pathways. **(A)** Western blot experiments were performed to detect p-AKT, p-SHP2, p-DNMT3B, P53 and MYH11 protein banding plots and relative protein expression statistics in Normoxic group, Hypoxic group, Normoxic+MK-2206 group, Hypoxic+MK-2206 group, Normoxic+Batoprotafib group and Hypoxic+Batoprotafib group; **(B)** Methylation-qPCR assay was performed to detect the methylation levels of P53 and MYH11 in Normoxic group, Hypoxic group, Normoxic+MK-2206 group, Hypoxic+MK-2206 group, Normoxic+Batoprotafib group and Hypoxic+Batoprotafib group; **(C)** RT-qPCR experiments were performed to detect the relative mRNA expression of P53β and Δ133P53 in Normoxic group, Hypoxic group, Normoxic+MK-2206 group, Hypoxic+MK-2206 group, Normoxic+Batoprotafib group and Hypoxic+Batoprotafib group Statistical plots of the amount of P53β and Δ133P53. Data were expressed as mean ± standard deviation. N = 3; *P<0.05; **P<0.01; ^ns^P>0.05: There was no significant difference.

Our previous experimental results demonstrated that overexpression of CBFβ-MYH11 could inhibit the expression of hypoxia-inducible factor 1α (HIF1α). Given that the hypoxic microenvironment is known to promote cancer progression and suppress p53 expression (19), we next aimed to investigate whether AKT and SHP2 play pivotal roles in this context. Huh-7 carcinoma cells were cultured synchronously in normoxic and hypoxic environments, with the addition of AKT and SHP2 inhibitors under hypoxia. Western Blot results showed that compared to normoxic conditions, the expression levels of p-AKT, p-SHP2, and p-DNMT3B were significantly increased in the hypoxic group, while the expression levels of P53 and CBFβ-MYH11 were significantly decreased. Addition of AKT inhibitor MK-2206 in both normoxic and hypoxic environments led to a significant decrease in p-AKT and p-DNMT3B expression levels with no difference between the two groups; compared to normoxic conditions, the expression levels of p-SHP2 significantly increased in the hypoxic group, while the expression levels of P53 and MYH11 decreased. The addition of SHP2 inhibitor Batoprotafib in both normoxic and hypoxic environments resulted in a significant decrease in p-SHP2 expression levels with no difference between the two groups; compared to normoxic conditions, the expression levels of p-AKT and p-DNMT3B increased in the hypoxic group, while the expression levels of P53 and MYH11 decreased significantly (**Figure 4A**).

Subsequent bisulfite qPCR experiments showed a significant increase in the methylation levels of P53 and CBFβ-MYH11 in the hypoxic group compared to the normoxic group. The addition of AKT inhibitor MK-2206 in both normoxic and hypoxic environments resulted in no difference in the methylation levels of P53 and CBFβ-MYH11 between the two groups. The addition of SHP2 inhibitor Batoprotafib in both normoxic and hypoxic environments led to a slight increase in the methylation levels of P53 and CBFβ-MYH11 compared to the normoxic group (**Figure 4B**).

RT-qPCR showed that the relative mRNA level of P53β was significantly lower in the hypoxic group than in the normoxic group, while the relative mRNA expression of Δ133P53 was significantly higher in the hypoxic group than in the normoxic group; after the addition of MK-2206, the mRNA expression of P53β in the hypoxic group and normoxic group was significantly higher, and the mRNA expression of Δ133P53 was significantly lower. However, the relative mRNA level of P53β was significantly lower in hypoxia than in normoxia, and there was no significant difference in the mRNA expression of Δ133P53. After the addition of Batoprotafib, the mRNA expression of P53β in the hypoxic and normoxic groups was significantly higher, and the relative mRNA level of P53β in the hypoxic group was significantly lower than that in the normoxic group; whereas the mRNA expression of Δ133P53 was significantly lower, and that in the hypoxic group was higher than that in the normoxic group (**Figure 4C**).

The above results suggested that AKT and SHP2 under hypoxic conditions jointly affect DNMT3B-mediated methylation changes of P53 and CBFβ-MYH11.

### 3.5 CBFβ-MYH11 mediated the migration and invasion capacity and angiogenesis ability of hepatocellular carcinoma in a low oxygen environment through the AKT and SHP2 pathways

**Figure 5:**
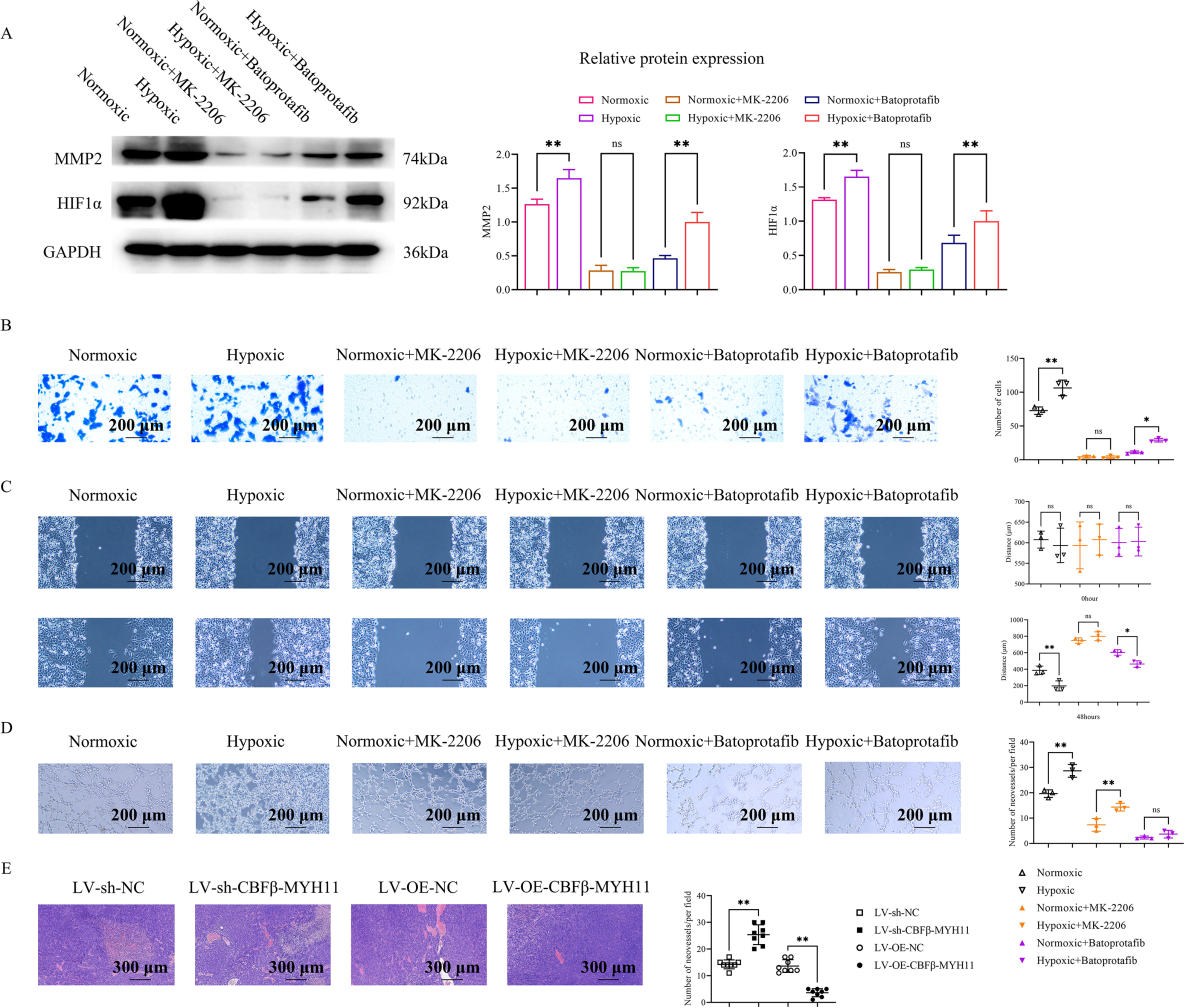
Migration, invasion, and angiogenesis of hepatocellular carcinoma under hypoxia induced by CBFβ-MYH11 through AKT and SHP2 pathways. **(A)** Western blot experiments were performed to detect the strip maps of MMP2 and HIF1α in the Normoxic group, Hypoxic group, Normoxic+MK-2206 group, Hypoxic+MK-2206 group, Normoxic+Batoprotafib group, and Hypoxic+Batoprotafib group and the relative protein expression statistics; GAPDH as control protein; N = 3; **(B)** Graphs of experimental results of Transwell assay to detect the cell migration ability of Normoxic group, Hypoxic group, Normoxic+MK-2206 group, Hypoxic+MK-2206 group, Normoxic+Batoprotafib group, and Hypoxic+Batoprotafib group as well as statistical graphs of the number of migrated cells; N = 3; **(C)** Plots of experimental results of cell scratch assay to detect cell migration ability of Normoxic group, Hypoxic group, Normoxic+MK-2206 group, Hypoxic+MK-2206 group, Normoxic+Batoprotafib group, and Hypoxic+Batoprotafib group as well as statistical plots of cell spacing; N = 3; **(D)** Graphs of the experimental results of Angiogenesis experiment to detect the angiogenic ability of Normoxic group, Hypoxic group, Normoxic+MK-2206 group, Hypoxic+MK-2206 group, Normoxic+Batoprotafib group, and Hypoxic+Batoprotafib group, as well as statistical graphs of the number of blood vessels; N = 3; **(E)** The results of HE staining experiments were plotted as well as the statistics of the number of blood vessels. N = 8; Data were expressed as mean ± standard deviation. **P<0.01; ^ns^P>0.05: There was no significant difference.

We synchronized Huh-7 hepatocellular carcinoma cells for cultivation in both normoxic and hypoxic environments, with the addition of AKT inhibitor and SHP2 inhibitor in the hypoxic environment. Western Blot results showed a significant increase in the expression of MMP2 and HIF1α in the hypoxic group compared to the normoxic group. The expression of MMP2 and HIF1α was significantly decreased in both groups when AKT inhibitor MK-2206 was added in both normoxic and hypoxic environments, and there was no difference between the two groups. However, when the SHP2 inhibitor Batoprotafib was added in both normoxic and hypoxic environments, the expression of MMP2 and HIF1α was significantly decreased in both groups. However, MMP2 and HIF1α protein expression was significantly higher in the hypoxic group relative to the normoxic group (**Figure 5A**).

Transwell results showed that the number of cells passing through the matrix gel increased significantly in the hypoxic group compared to the normoxic group. When AKT inhibitor MK-2206 was added simultaneously in both normoxic and hypoxic environments, the number of cells passing through the matrix gel decreased significantly in both the normoxic and hypoxic groups with no difference between the two groups. Likewise, when SHP2 inhibitor Batoprotafib was added simultaneously in both normoxic and hypoxic environments, the number of cells passing through the matrix gel increased significantly in the hypoxic group compared to the normoxic group (**Figure 5B**).

Scratch results showed that the scratch width of the hypoxic group decreased significantly compared to the normoxic group. When AKT inhibitor MK-2206 was added simultaneously in both normoxic and hypoxic environments, the scratch width decreased significantly in both the normoxic and hypoxic groups with no difference between the two groups. Similarly, when SHP2 inhibitor Batoprotafib was added simultaneously in both normoxic and hypoxic environments, the scratch width of the hypoxic group decreased significantly compared to the normoxic group (**Figure 5C**).

Angiogenesis results showed that the number of blood vessels increased significantly in the hypoxic group compared to the normoxic group. When AKT inhibitor MK-2206 was added simultaneously in both normoxic and hypoxic environments, the number of blood vessels increased significantly in both the normoxic and hypoxic groups. Conversely, when SHP2 inhibitor Batoprotafib was added simultaneously in both normoxic and hypoxic environments, the number of blood vessels in the hypoxic group decreased significantly compared to the normoxic group with no difference between the two groups (**Figure 5D**). We then observed the number of blood vessels in the tumors of nude mice through *in vivo* HE experiments. The results showed that the number of blood vessels in the LV-sh-CBFβ-MYH11 group was significantly higher than that in the LV-sh-NC group. The number of blood vessels in the LV-OE-CBFβ-MYH11 group was significantly lower than that in the LV-OE-NC group (**Figure 5E**). The above results illustrated that CBFβ-MYH11 mediates the migratory invasive ability and angiogenic capacity of hepatocellular carcinoma in hypoxic environment through AKT and SHP2 pathways.

### 3.6 P53 mediated the antagonistic effect of low oxygen environment on the CBFβ-MYH11 and SHP2 pathways in hepatocellular carcinoma

**Figure 6:**
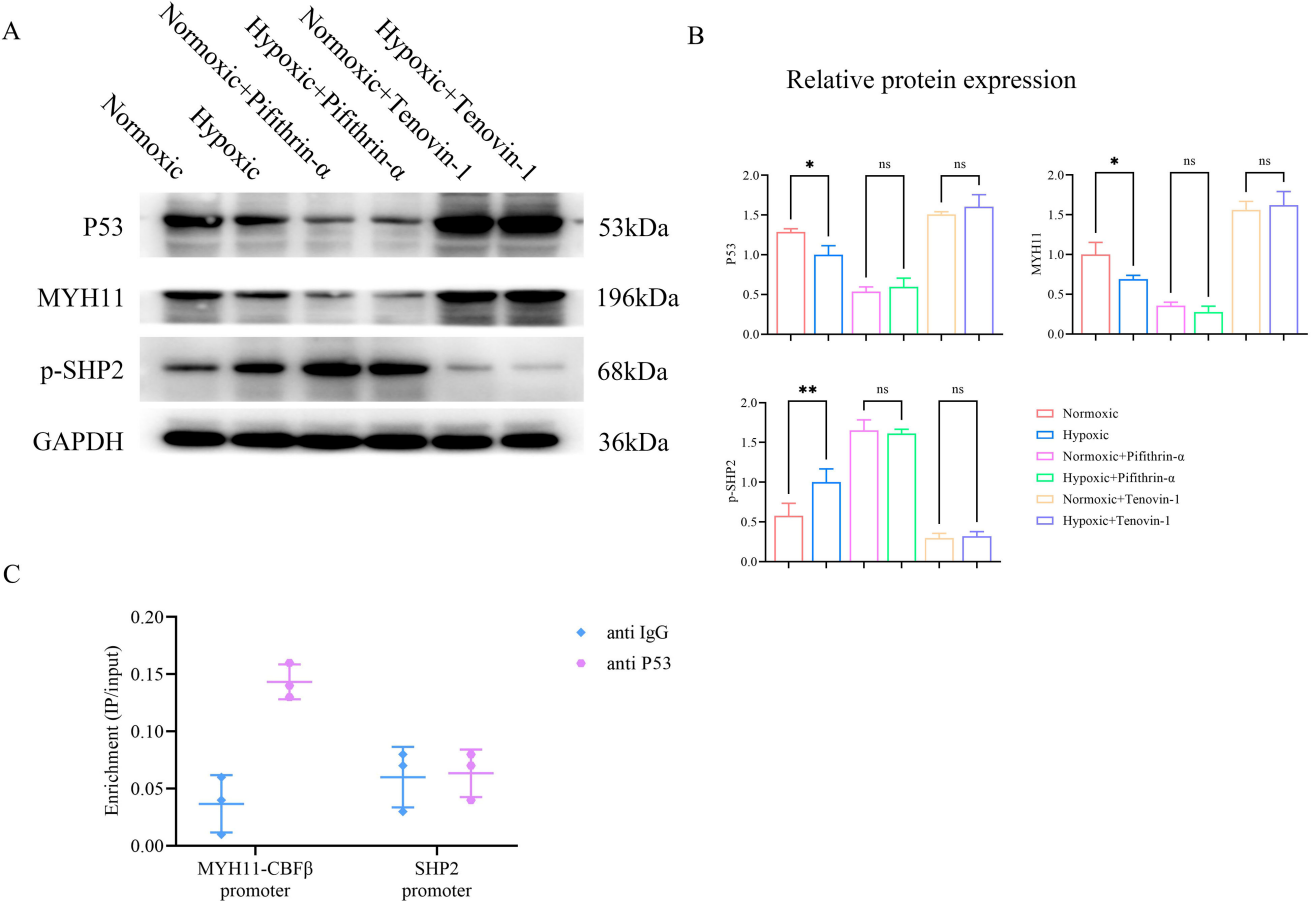
The role of P53 in antagonizing the CBFβ-MYH11 and SHP2 pathways under low oxygen conditions. **(A, B)** Western blot experiments were performed to detect P53, MYH11 and p-SHP2 bar graphs as well as relative protein expression statistics. in Normoxic, Hypoxic, Normoxic+Pifithrin-a, Hypoxic+Pifithrin-a, Normoxic+Tenovin-1 and Hypoxic+Tenovin-1 groups; **(C)** Chromatin immunoprecipitation-qPCR demonstrated that P53 could enrich the CBFβ-MYH11 promoter, with a smaller effect on the SHP2 promoter. Data were expressed as mean ± standard deviation. *P<0.05; **P<0.01; ^ns^P>0.05: There was no significant difference.

Although the roles of CBFβ-MYH11 *in vivo* and *in vitro* are known, how P53 affects CBFβ-MYH11 and SHP2 remains unclear. Through experiments with P53 inhibitor Pifithrin-α and P53 activator Tenovin-1, Western Blot results showed that the expression levels of P53 and MYH11 in the hypoxic group decreased significantly compared to the normoxic group, while the expression level of p-SHP2 increased significantly. When P53 inhibitor Pifithrin-α was added simultaneously in both normoxic and hypoxic environments, the expression levels of P53 and MYH11 decreased significantly in both the normoxic and hypoxic groups with no difference between them, while the expression level of p-SHP2 increased significantly with no difference between the two groups. Conversely, when P53 activator was added simultaneously in both normoxic and hypoxic environments, the expression levels of P53 and MYH11 increased significantly in both the normoxic and hypoxic groups with no difference between them, while the expression level of p-SHP2 decreased significantly with no difference between the two groups (**Figures 6A, B**). By using the chromatin immunoprecipitation-qPCR method, we examined the binding of P53 to the downstream promoter. The results showed a significant increase in the enrichment level of the CBFβ-MYH11 promoter bound by P53/IP compared to the negative control IgG, but the enrichment level of the SHP2 promoter bound by P53/IP was not as pronounced (**Figure 6C**). The above results illustrated the antagonistic effect of P53-mediated hypoxic environment on CBFβ-MYH11 and SHP2 pathways in hepatocellular carcinoma.

### 3.7 RUNX1 could counteract the tumor-suppressive effect of CBFB-MYH11 in hepatocellular carcinoma

**Figure 7:**
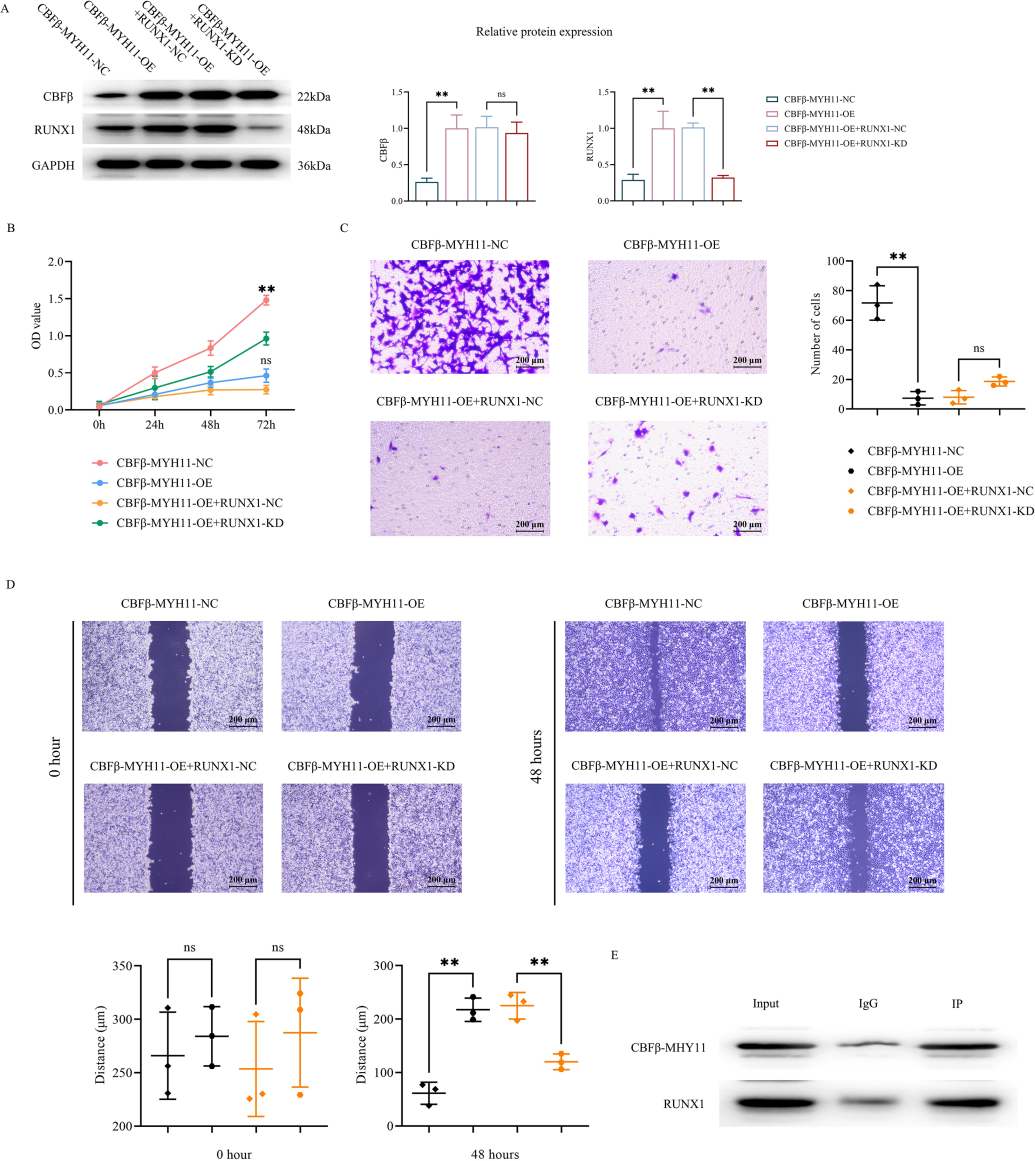
The rescue of the tumor suppressive effect of CBFβ-MYH11 on hepatocellular carcinoma by RUNX1 under low oxygen conditions. **(A)** Western blot experiments were performed to detect the bar graphs of CBFβ and RUNX1 as well as the statistical graphs of relative protein expression in the CBFβ-MYH11-NC group, CBFβ-MYH11-OE group, CBFβ-MYH11-OE+RUNX1-NC group and CBFβ-MYH11-OE+RUNX1-KD group; **(B)** Statistical graphs of OD values of CBFβ-MYH11-NC group, CBFβ-MYH11-OE group, CBFβ-MYH11-OE+RUNX1-NC group and CBFβ-MYH11-OE+RUNX1-KD group detected by CCK-8 assay; **(C)** Graphs of the results of Transwell assays for the CBFβ-MYH11-NC group, CBFβ-MYH11-OE group, CBFβ-MYH11-OE+RUNX1-NC group and CBFβ-MYH11-OE+RUNX1-KD group, as well as statistics of the number of migrating cells; **(D)** Plot of the results of the cell scratch assay to detect CBFβ-MYH11-NC group, CBFβ-MYH11-OE group, CBFβ-MYH11-OE+RUNX1-NC group and CBFβ-MYH11-OE+RUNX1-KD group as well as the statistical plot of the spacing of cell scratches; **(E)** CO-IP experiments confirmed the interaction between CBFβ-MYH11 and RUNX1. Data were expressed as mean ± standard deviation. **P<0.01; ^ns^P>0.05: There was no significant difference.

Western Blot experiments showed that in hepatocellular carcinoma cell lines, Huh-7 was cultivated in a 1% low oxygen environment. Compared to the LV-OE-NC group, the LV-OE-CBFβ-MYH11 group showed a significant increase in CBFβ protein expression, as well as in RUNX1 protein expression. Consistently, the LV-OE-CBFβ-MYH11+LV-KD-NC group exhibited a significant increase in CBFB-MYH11 protein expression, along with a significant increase in RUNX2 protein expression. Compared to the LV-OE-CBFβ-MYH11+LV-OE-NC group, the LV-OE-CBFβ-MYH11+LV-KD-RUNX1 group showed a significant increase in CBFβ protein expression, a significant decrease in RUNX1 protein expression, and a subtle change in protein expression (**Figure 7A**). CCK-8 experiments showed that in hepatocellular carcinoma cell lines, Huh-7 was cultivated in a 1% low-oxygen environment. Compared to the LV-OE-NC group, the LV-OE-CBFβ-MYH11 group exhibited a significant decrease in the OD value at 450nm, as did the LV-OE-CBFβ-MYH11+LV-KD-NC group. Compared to the LV-OE-CBFβ-MYH11+LV-OE-NC group, the LV-OE-CBFβ-MYH11+ LV-KD-RUNX1 group showed a significant increase in the OD value at 450nm for CBFB-MYH11 (**Figure 7B**). Transwell experiments showed that in hepatocellular carcinoma cell lines, Huh-7 was cultivated in a 1% low-oxygen environment. Compared to the LV-OE-NC group, the LV-OE-CBFβ-MYH11 group showed a significant decrease in the number of migrating cells, as did the LV-OE-CBFβ-MYH11+ LV-KD-NC group. Compared to the LV-OE-CBFβ-MYH11+LV-OE-NC group, the LV-OE-CBFβ-MYH11+LV-KD-RUNX1 group showed a significant increase in the number of migrating cells (**Figure 7C**). Scratch experiments showed that in hepatocellular carcinoma cell lines, Huh-7 was cultivated in a 1% low-oxygen environment. Compared to the LV-OE-NC group, the LV-OE-CBFβ-MYH11 group showed a significant increase in scratch width, as did the LV-OE-CBFβ-MYH11+LV-KD-NC group. Compared to the LV-OE-CBFβ-MYH11+LV-OE-NC group, the LV-OE-CBFβ-MYH11+ LV-KD-RUNX1 group showed a significant decrease in scratch width (**Figure 7D**). Co-IP experiments showed that in hepatocellular carcinoma cell lines, Huh-7 was cultivated in a 1% low-oxygen environment. Subsequent Co-IP detection revealed an interaction between CBFB-MYH11 and RUNX1 proteins (**Figure 7E**). The above results indicated that RUNX1 counteracted the tumor suppressor effect of CBFB-MYH11 on hepatocellular carcinoma.

### 3.8 DNMT3B interacts with MYH11 and negatively regulates its expression, and hypoxic microenvironment induces hypermethylation of the MYH11 promoter

**Figure 8:**
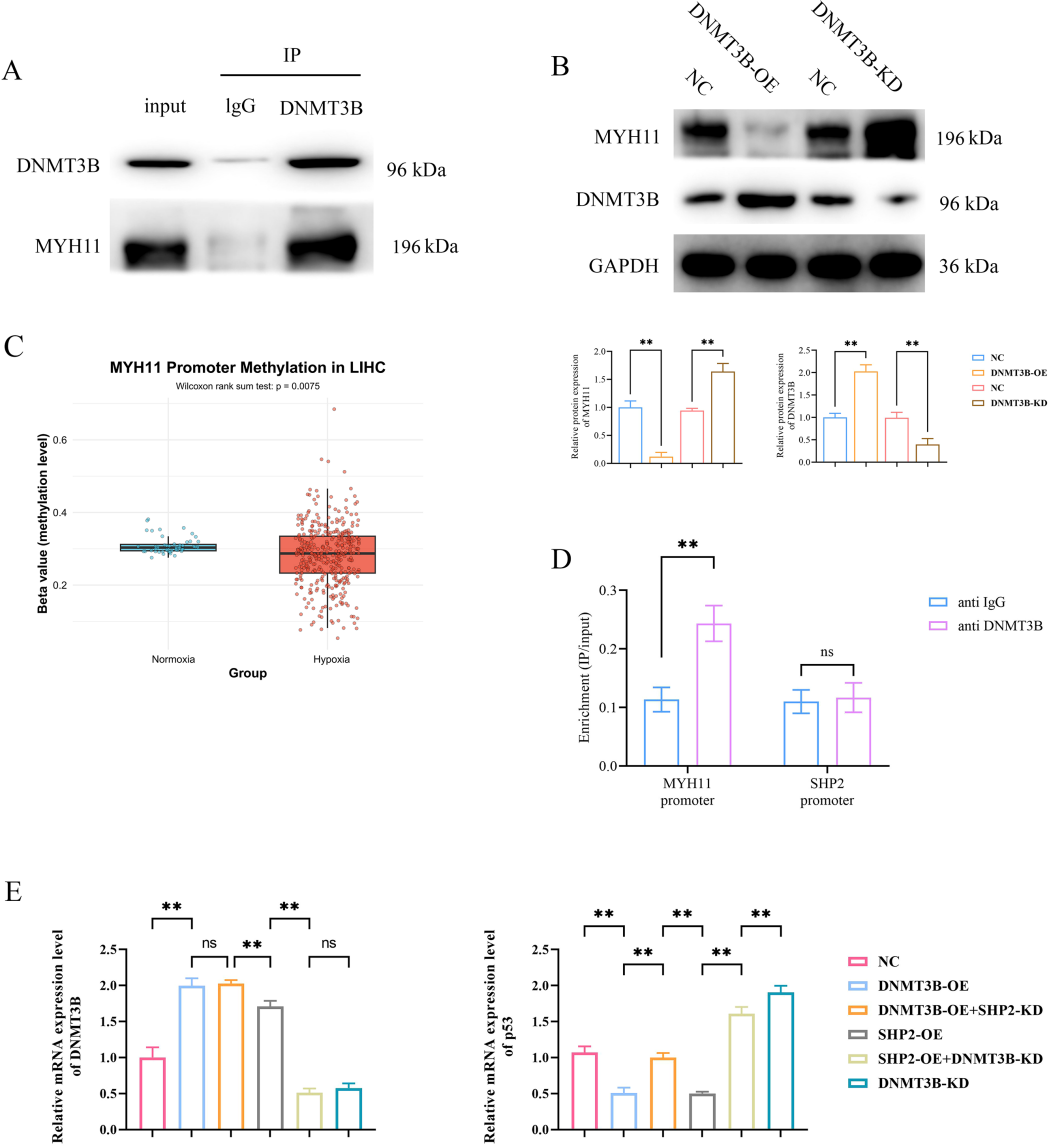
DNMT3B Interacts with MYH11 and Negatively Regulates Its Expression, and Hypoxic Microenvironment Induces Hypermethylation of the MYH11 Promoter. **(A)** CO-IP experiments confirmed the interaction between DNMT3B and MYH11; **(B)** Western blot experiments were performed to detect the bar graphs of DNMT3B and MYH11 as well as the statistical graphs of relative protein expression in the NC group, DNMT3B-OE group, NC group and DNMT3B-KD group; **(C)** The box plot shows the distribution of average methylation β-values of the MYH11 promoter region in LIHC tumor samples stratified by the median hypoxia score; **(D)** ChIP-qPCR analysis of the binding of DNMT3B to the MYH11 and SHP2 promoters; **(E)** qPCR detection of relative mRNA expression levels in the NC group, DNMT3B-OE group, DNMT3B-OE+SHP2-KD group, SHP2-OE group, SHP2-OE+DNMT3B-KD group, and DNMT3B-KD group. Data were expressed as mean ± standard deviation. **P<0.01.

To investigate the regulatory effect of DNMT3B on MYH11 protein levels, co-immunoprecipitation (Co-IP) assays revealed that DNMT3B and MYH11 interact with each other (**Figure 8A**). Western blot analysis showed that, compared with the NC group, the relative expression level of MYH11 was significantly decreased while that of DNMT3B was markedly increased in the DNMT3B-OE group. In contrast, the relative expression level of MYH11 was significantly upregulated and that of DNMT3B was notably downregulated in the DNMT3B-KD group relative to the NC group(**Figure 8B**). Given the close association between tumor hypoxia and alterations in DNA methylation, we further analyzed the correlation between hypoxia status and MYH11 promoter methylation levels. As shown in the figure, the average methylation level of the MYH11 promoter was significantly higher in the hypoxia group than in the normoxia group (Wilcoxon rank-sum test, P = 0.0075) (**Figure 8C**). We employed ChIP-qPCR to examine the binding of DNMT3B to the MYH11 and SHP2 promoters. The results showed that, compared with the negative control IgG group, the enrichment of the MYH11 promoter in DNMT3B immunoprecipitates was significantly increased, whereas enrichment at the SHP2 promoter was not pronounced (**Figure 8D**). Quantitative PCR (qPCR) was performed to detect the relative mRNA levels of p53 and DNMT3B. The results indicated that, compared with the NC group, the relative mRNA level of p53 was significantly decreased and that of DNMT3B was significantly increased in the DNMT3B-OE group; compared with the DNMT3B-OE group, the relative mRNA level of p53 was significantly increased while that of DNMT3B showed no significant difference in the DNMT3B-OE+SHP2-KD group; compared with the DNMT3B-OE+SHP2-KD group, the relative mRNA level of p53 was significantly decreased and that of DNMT3B was significantly decreased in the SHP2-OE group; compared with the SHP2-OE group, the relative mRNA level of p53 was significantly increased and that of DNMT3B was significantly decreased in the SHP2-OE+DNMT3B-KD group; compared with the SHP2-OE+DNMT3B-KD group, the relative mRNA level of p53 was significantly increased while that of DNMT3B showed no significant difference in the DNMT3B-KD group (**Figure 8E**). These results indicated that DNMT3B interacts with MYH11 and negatively regulates its expression, that the hypoxic microenvironment induces hypermethylation of the MYH11 promoter via DNMT3B, and that DNMT3B cooperates with SHP2 to suppress P53 expression.

### 3.9 Validation of hypoxia signaling axis clinical relevance in independent HCC cohorts

#### Consistent gene correlations across three cohorts

**Figure 9:**
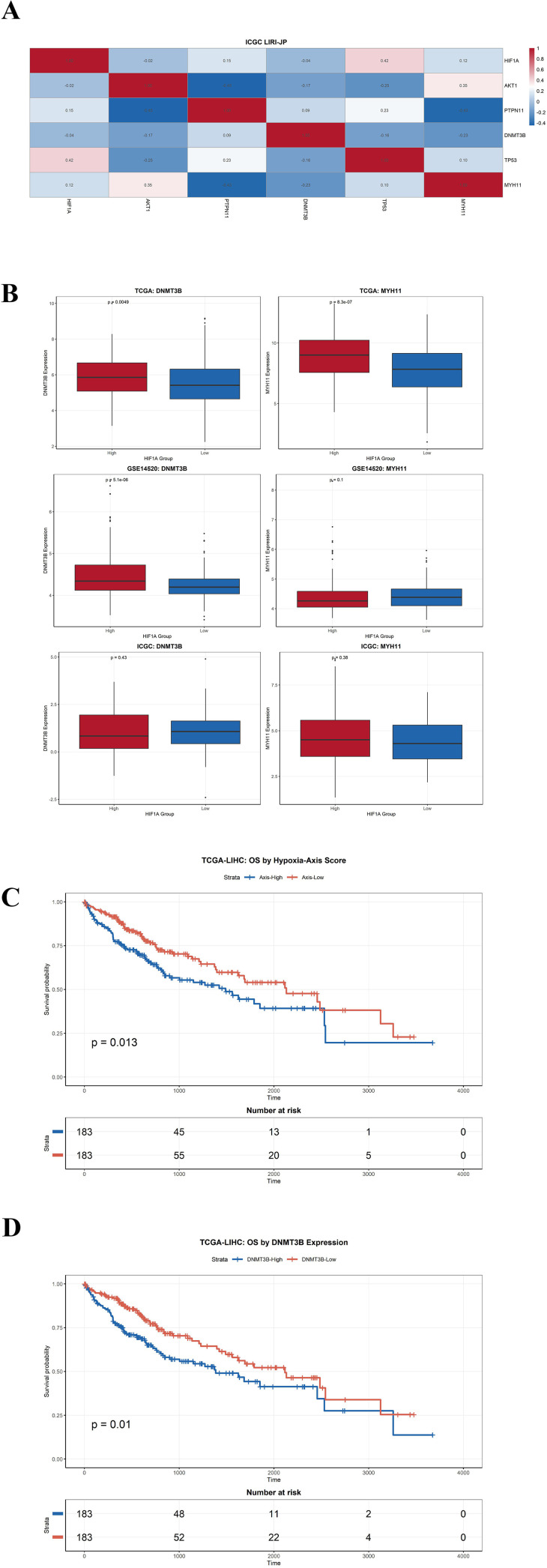
Validation of Hypoxia Signaling Axis Clinical Relevance in Independent HCC Cohorts. **(A)** Spearman correlation matrix of pivotal axis genes across three independent HCC cohorts; **(B)** Differential expression of DNMT3B and MYH11 between HIF1A high- and low-expression groups; **(C)** Kaplan-Meier overall survival curves based on composite Axis Score in the TCGA-LIHC cohort; **(D)** Kaplan-Meier overall survival curves stratified by DNMT3B expression level in the TCGA-LIHC cohort.

Spearman correlation matrices of six axis-related genes presented highly consistent correlation patterns in TCGA-LIHC, GSE14520 and ICGC LIRI-JP cohorts (**Figure 9A**). Negative correlations between DNMT3B and MYH11 were observed in all three cohorts (TCGA: r = -0.097; GSE14520: r = -0.295; ICGC: r = -0.226). DNMT3B was negatively correlated with TP53 in the ICGC cohort (r = -0.163). Positive correlations between HIF1A and DNMT3B were found in TCGA (r = 0.185) and GSE14520 (r = 0.271). The correlation trends were consistent with the regulatory mechanism that hypoxia upregulates DNMT3B to transcriptionally silence P53 and MYH11, providing population-level transcriptomic evidence for this signaling axis.

#### Expression differences of DNMT3B and MYH11 Stratified by HIF1A Level

Stratified by the median HIF1A expression level, DNMT3B tended to be highly expressed while MYH11 was downregulated in the Hypoxia-High group across all cohorts. Statistical differences were detected in the TCGA cohort (DNMT3B: P = 0.003; MYH11: P < 0.001) (**Figure 9B**). The findings verified that hypoxia status marked by HIF1A is associated with elevated DNMT3B and reduced MYH11, confirming the coordinated transcriptional regulation of axis molecules.

#### Correlation between axis score and HCC patient prognosis

The composite Axis Score calculated based on Z-scores of the six genes exhibited powerful prognostic stratification capability in the TCGA-LIHC cohort (**Figure 9C**). Patients in the Axis-High group had significantly poorer overall survival compared with those in the Axis-Low group (log-rank P = 0.01). Univariate Cox regression analysis revealed that continuous Axis Score was positively correlated with mortality risk (HR = 1.125, 95% CI: 1.051–1.203, P < 0.001). High DNMT3B expression also indicated shortened survival time (**Figure 9D**), which was in accordance with the prognostic tendency of Axis Score. Collectively, the activated hypoxia signaling axis serves as an independent adverse prognostic risk factor for HCC patients.

### 3.10 Mechanistic model of the tumor-suppressive role of CBFβ-MYH11 in hepatocellular carcinoma

**Figure 10:**
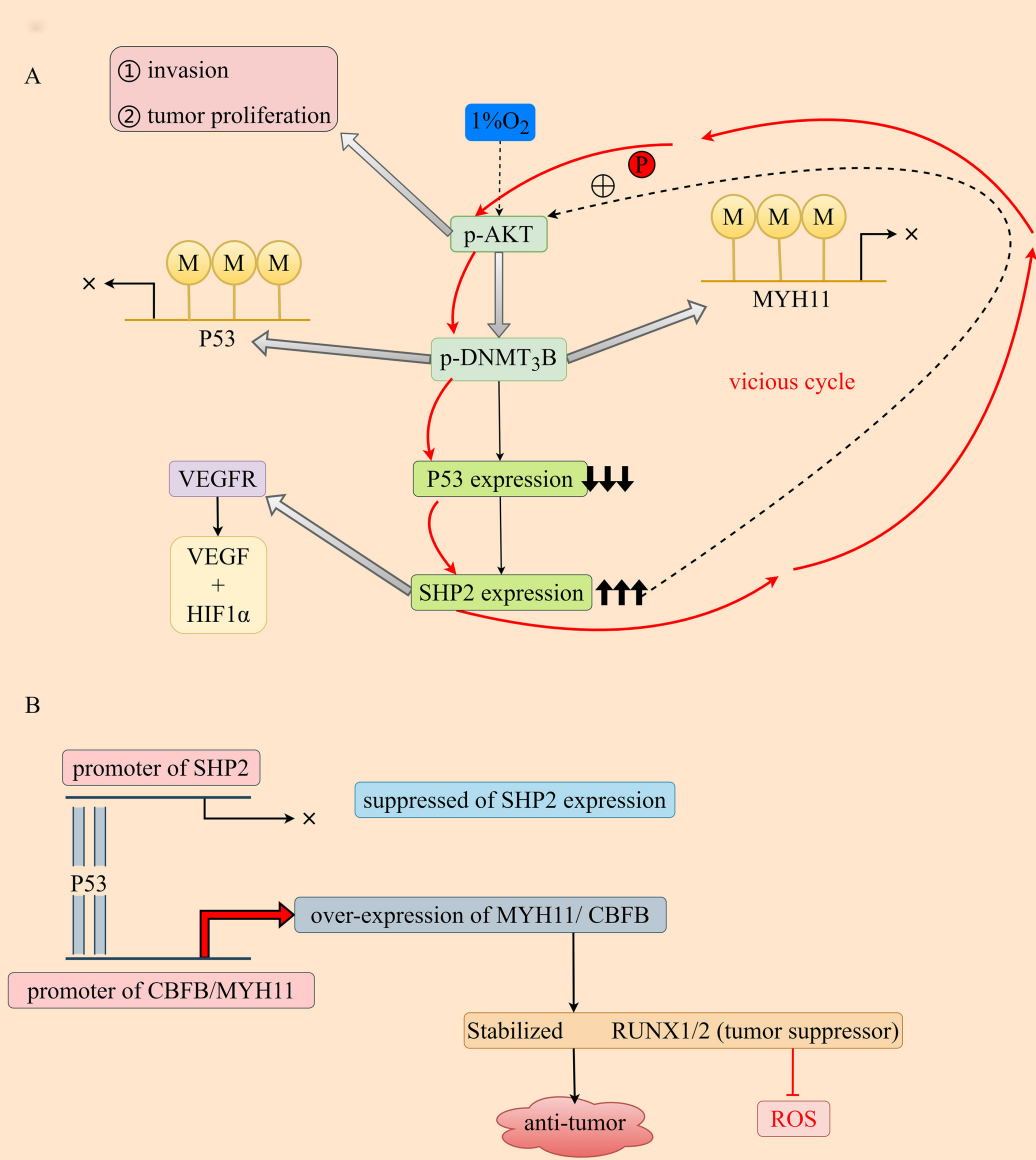
Schematic representation of the tumor suppressive mechanism of CBFβ-MYH11 in hepatocellular carcinoma. **(A)** Under low oxygen conditions, activation of AKT promotes cell survival and growth, while the activity of DNMT3B is regulated, leading to changes in DNA methylation. Overexpression of DNMT3B inhibits the function of P53, promoting tumor development; **(B)** SHP2 interacts with P53, modulating the activity of AKT through phosphorylation. Aberrant expression and mutations of CBFB-MYH11 are associated with certain cancers.

In a low-oxygen environment, AKT was activated, promoting cell survival and growth. The activation of the AKT signaling pathway regulated the activity of DNMT3B, affecting DNA methylation and cell functions. Overexpression of DNMT3B inhibited P53 function, promoting tumor initiation and progression. SHP2 interacted with P53, regulating AKT activity, and influencing the AKT signaling pathway through phosphorylation. Aberrant expression and mutations of CBFβ-MYH11 were associated with some types of cancer (**Figures 10A, B**). The original membranes for this study were supplemented in **Supplementary File 1**, and the original data were supplemented in **Supplementary File 2**.

## 4 Discussion

HCC is one of the most common cancers, and its high rate of invasiveness and ability to cause death are two of the main reasons why. Nevertheless, the molecular pathways that facilitate hypoxia-induced invasion and migration of HCC cells are not yet fully elucidated (20, 21). Hypoxia, defined as an inadequate oxygen supply to fulfill physiological requirements, significantly modifies gene expression, especially in the tumor microenvironment. AKT, also known as Protein Kinase B, is an important signaling protein that is often turned on when there is not enough oxygen. It controls many different cellular functions (22). SHP2 (Src Homology 2 domain-containing Protein Tyrosine Phosphatase-2) is an important part of the complex network of signaling that happens inside cells. This non-receptor tyrosine phosphatase is involved in many signaling pathways that promote tumor growth, invasion, and metastasis. Recent studies have clarified the mechanism by which hypoxia stimulates the increase of AKT expression. The strategic use of AKT inhibitors was shown to lower AKT expression, whereas SHP2 inhibitors did the opposite and raised it. This indicates that SHP2 may be involved in AKT-mediated effects. But in an interesting twist, stopping SHP2 from working made it more active. And stopping AKT from working also caused SHP2 levels to go up. These results suggest that there is a complex interplay going on between these molecules. Furthermore, hypoxia alters DNA methylation and other epigenetic processes and switches the epigenetic landscape (23).

DNA Methyltransferase 3B (DNMT3B) is an important element of the genome that is needed to produce new methylation patterns throughout cell differentiation and embryonic development. It safeguards gene expression by significantly influencing the activation or deactivation of genetic elements. Furthermore, our data demonstrated that DNMT3B directly binds to MYH11 and downregulates its expression. Co-immunoprecipitation assays confirmed the physical interaction between DNMT3B and MYH11. DNMT3B overexpression led to a significant decrease in MYH11 protein levels as determined by western blot analysis, while DNMT3B knockdown largely rescued MYH11 expression. In addition, the hypoxic microenvironment caused a significant hypermethylation of the MYH11 promoter, with a higher mean methylation level in hypoxic tumor samples compared to normoxic controls. ChIP-qPCR experiments further confirmed the strong enrichment of DNMT3B in the promoter region of MYH11, indicating that hypoxia-induced upregulation of DNMT3B mediates MYH11 silencing via promoter hypermethylation.

In the present study, we confirmed a stable negative correlation between DNMT3B and MYH11 in three independent HCC cohorts, including TCGA-LIHC, GSE14520, and ICGC LIRI-JP. DNMT3B was higher and MYH11 was lower in the HIF1A-high subgroup. The composite Axis Score constructed based on hub genes was able to effectively stratify patient prognosis. High Axis Score was associated with shortened overall survival and was an independent adverse prognostic factor, which clinically supported the mechanism that hypoxia upregulates DNMT3B to induce MYH11 silencing. This study demonstrates the hypoxia-induced increase of the DNMT3B expression suggesting a link between oxygen deprivation and gene regulation by methylation (24). The P53 protein is a major tumor suppressor that checks cells for problems and triggers a number of protective responses, such as stopping the cell cycle, repairing DNA, inducing apoptosis or causing cells to age in response to DNA damage or other signs of cellular distress (25). One of its major purposes is to stop the spread of abnormal cells that can cause cancer. The complexities of P53 function are very much related to methylation of CpG islands, a biological mechanism that can modify the expression of P53 controlled genes. This DNA methylation often prevents genes from working, adding to the complexity of the regulation. It is known in oncology that hypermethylation can block the P53-mediated repression of certain genes, thus favoring carcinogenesis. DNMT3B (DNA Methyltransferase 3B) plays a major role in this context by increasing methylation of P53 target genes (26). Such a modulatory effect suggests that DNMT3B might accidentally promote silencing of genes that otherwise, under the strict supervision of P53, suppress the growth of cancer cells. The complex relationship between methylation and gene expression under DNMT3B’s impact shows how complicated gene regulation systems are for stopping tumors and keeping cells healthy (27). Importantly, our rescue experiments further revealed that DNMT3B cooperates with SHP2 to suppress P53 expression.

Also, the levels of methylation in CBFβ-MYH11 were examined and shown to rise in low-oxygen situations, just as P53 methylation. CBFβ-MYH11 (Core Binding Factor Beta-Myosin Heavy Chain 11) encodes a smooth muscle myosin heavy chain, and mutations in this gene are linked to many illnesses and several cancer types. CBFβ-MYH11 may not directly respond to hypoxia; nevertheless, the signaling networks activated under hypoxic environments may modify gene expression patterns, functional status, and behavior of smooth muscle cells, thereby influencing CBFβ-MYH11 expression or function. In response to hypoxia, cells may trigger angiogenesis to improve oxygen delivery, potentially activating or reprogramming smooth muscle cells, which are essential parts of the vascular wall, and affecting CBFβ-MYH11 expression (28).

In some malignancies, unregulated AKT signaling and impaired SHP2 are associated with metastasis and invasion. The functional alterations in CBFβ-MYH11 have a substantial impact on the tumor microenvironment. This work shows that the AKT signaling pathway increases the expression of CBFβ-MYH11, which is also thought to be the case for SHP2. Inhibiting AKT signaling notably diminishes DNMT3B expression and lowers the methylation levels of P53 and MYH. Hypoxia encourages cells to move, invade, and grow, while AKT and P53 inhibitors lessen these effects. They also fight against changes in methylation caused by DNMT3B and show how important DNMT3B is in methylation dynamics. Higher expression of CBFβ-MYH11 is correlated with decreased motility and invasion of hepatocellular carcinoma (HCC) cells. In the nude mouse xenograft assay, higher expression of CBFβ-MYH11 may inhibit tumor formation and angiogenesis. The study indicates that hypoxia affects P53 regulation through the AKT/DNMT3B/SHP2 pathway, potentially suppressing tumor development. This intricate molecular interplay offers a nuanced understanding of signaling networks and identifies potential therapeutic targets (29). The AKT/DNMT3B/P53 pathway acts on CBFβ-MYH11 to promote CBFβ-MYH11 expression, and CBFβ-MYH11 regulation of RUNX1 is not dependent on the AKT/DNMT3B/P53 pathway.

CBFβ-MYH11 can activate RUNX1 and increase the expression of RUNX1RUNX1 can inhibit oxidative stress and thus inhibit the expression of AKT and DNMT3B, which leads to an increase in the relative protein expression level of P53, and a decrease in the expression level of SHP2, which induces VEGF and HIF1α expression, and a decrease in the SHP2. SHP2 can induce the expression of VEGF and HIF1α, and the reduced expression level of SHP2 will also reduce the expression protein level of VEGF and HIF1α.VEGF enhances the migration and invasion ability of the cells, and participates in angiogenesis and increases the vascular permeability, and HIF1α plays an important role in tumor angiogenesis, and the reduction of the protein expression level of VEGF and HIF1α will result in the reduction of the migration, invasion, and angiogenesis of the hepatocellular carcinoma cells.

In essence, the collective data presented suggest a noteworthy characterization of CBFβ-MYH11 as a tumor suppressor within the context. It describes how CBFβ-MYH11 stops tumors from growing, moving, invading, and making new blood vessels. This is accomplished by inhibiting critical promotive factors, including MMP2, VEGF, and HIF1α. The study further emphasizes a significant finding: hypoxic circumstances facilitate the development of the illness. This process is mechanistically enabled by a methylation-dependent inhibition of two essential genes—P53 and CBFβ-MYH11. In this framework, AKT and SHP2 are recognized as pivotal entities that exert impact through the molecular mechanisms of DNMT3B. The detailed insights derived from this work illuminate novel avenues for therapeutic research and intervention, including the potential to impede or reverse the detrimental trajectory of HCC growth.

In hypoxia-associated hepatocellular carcinoma (HCC), we identify the actionable precision targets DNMT3B and SHP2 with clear translational potential in this study. The hypoxic microenvironment triggers the hypermethylation of MYH11 by activating DNMT3B expression via AKT, and inhibits P53. SHP2 acts as an upstream key regulator to synergistically promote DNMT3B function and potentiate hypoxia-mediated oncogenic effects. Therefore, the combination of DNMT3B and SHP2 targeting may be a promising strategy to overcome hypoxia-induced drug resistance and inhibit tumor progression. In addition, the HIF1A/AKT/SHP2/DNMT3B/P53/MYH11 axis score constructed in this study can distinguish HCC patients with high hypoxia and high DNMT3B/SHP2 expression, which can guide personalized therapy and enhance the clinical benefit rate. Future studies should include combination treatment in orthotopic HCC models, organoid-based drug sensitivity assays, and pharmacodynamic biomarker analysis in clinical specimens to promote the clinical translation of this combinatorial strategy.

Although the present study revealed the molecular mechanism by which CBFβ−MYH11 mediates P53 expression by regulating the AKT/DNMT3B/SHP2 pathway under hypoxic conditions, thereby inhibiting the progression of hepatocellular carcinoma (HCC), several limitations still exist in this study. First, the *in vivo* experiments were performed using a subcutaneous xenograft tumor model in immunodeficient nude mice, without establishing an orthotopic HCC model. This makes it difficult to simulate the actual tumorigenic and developmental microenvironment of clinical HCC, and the clinical translational value of the experimental results requires further validation. Second, the specific interaction sites among molecules in the pathway, as well as the downstream regulatory targets through which RUNX1 antagonizes the tumor−suppressive effect of CBFβ−MYH11, have not been fully elucidated. In addition, the crosstalk between this core pathway and other signaling pathways in the hypoxic tumor microenvironment has not been systematically investigated. Third, the detection of the CBFβ−MYH11 fusion transcript was performed only by RT−qPCR targeting the fusion junction. In the absence of validation by genomic DNA sequencing or long−read RNA sequencing, we cannot definitively confirm that this transcript originates from a classical chromosomal inversion rather than complex transcriptional events. Therefore, the endogenous genomic status of CBFβ-MYH11 in HCC needs to be clarified.

These limitations point to directions for future research. Further studies can be carried out by clinical samples, orthotopic tumor models, in-depth dissection of the details of molecular interactions, and the crosstalk regulatory mechanism between this pathway and the tumor microenvironment. This will help to refine the regulatory mechanism of this molecular pathway in HCC development and provide more reliable experimental evidence for targeted therapy of HCC.

## Data Availability

The raw data supporting the conclusions of this article will be made available by the authors, without undue reservation.
