# Supplementary material for: Hypoxia induced DNMT3B and SHP2 signaling promoted HCC via suppressing P53 and MYH11 protein expression
Source: Front Oncol. 2026 Jun 15;16:1794481. doi: 10.3389/fonc.2026.1794481 (PMC13311105; doi:10.3389/fonc.2026.1794481)
Supplement: Supplementary file 1 [file DataSheet1.pdf]

# Supplementary file 1: Original strip chart of western blot for this study

**Fig3A**

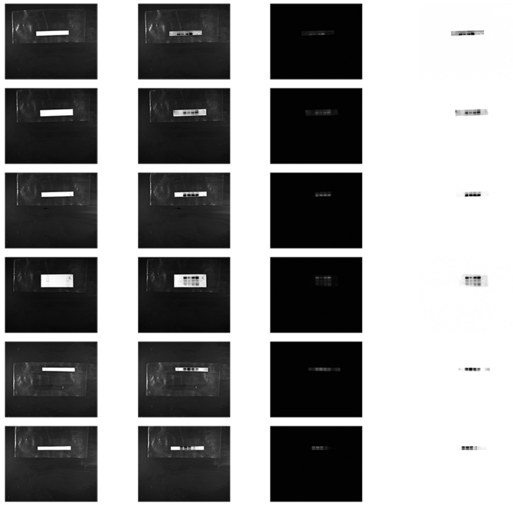

VEGF

MMP2

GAPDH

HIF1α

RUNX1

MYH11

**Fig4A**

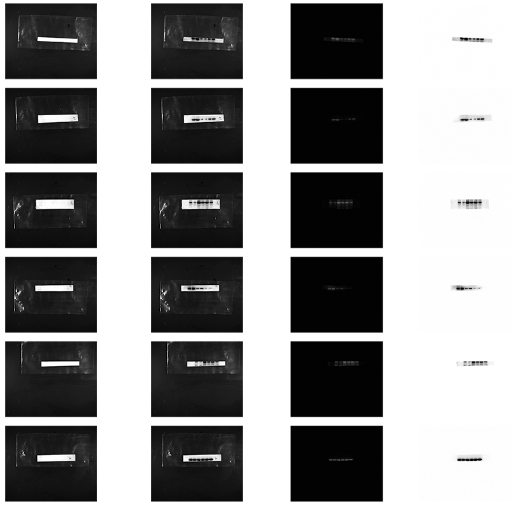

p-AKT

p-DNMT3B

P53

p-SHP2

MYH11

GAPDH

**Fig5A**

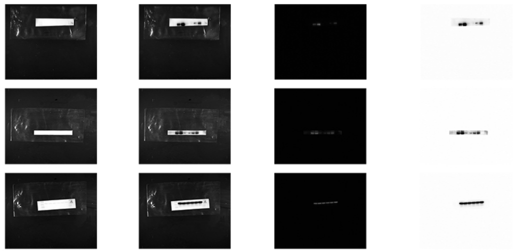

HIF1α

MMP2

GAPDH

**Fig6A**

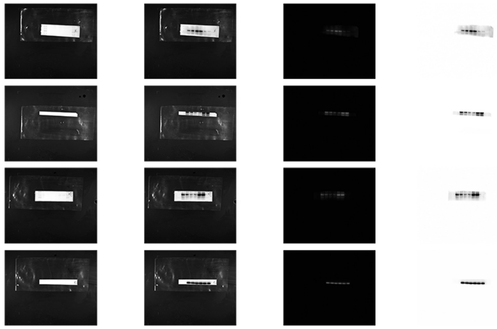

p-SHP2

MYH11

P53

GAPDH

**Fig7A**

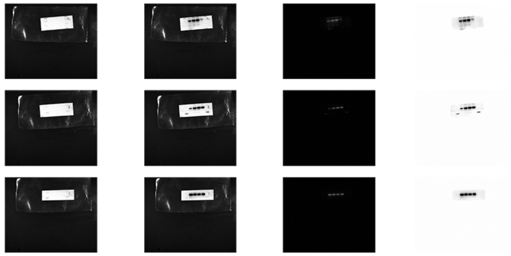

RUNX1

CBFβ

GAPDH

**Fig7F**

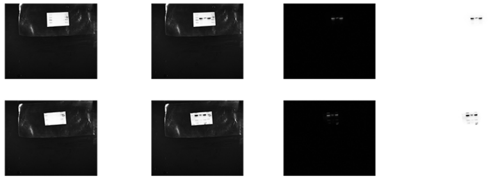

CBFβ-MYH11

RUNX1

**Fig8A**

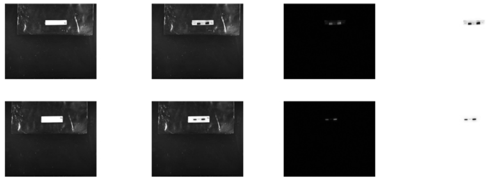

MYH11

DNMT3B

**Fig8B**

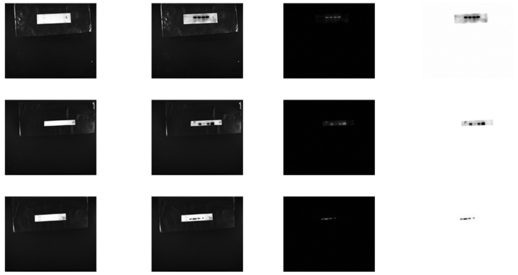

GAPDH

MYH11

DNMT3B
